# FAIMS Shotgun Lipidomics for Enhanced Class- and Charge-State Separation Complemented by Automated Ganglioside Annotation

**DOI:** 10.1021/acs.analchem.4c01313

**Published:** 2024-07-19

**Authors:** Katharina Hohenwallner, Leonida M. Lamp, Liuyu Peng, Madison Nuske, Jürgen Hartler, Gavin E. Reid, Evelyn Rampler

**Affiliations:** † Department of Analytical Chemistry, Faculty of Chemistry, University of Vienna, Vienna 1090, Austria; ‡ Vienna Doctoral School in Chemistry (DoSChem), University of Vienna, Vienna 1090, Austria; § Institute of Pharmaceutical Sciences, University of Graz, Graz 8010, Austria; ∥ School of Chemistry, University of Melbourne, Parkville, Victoria 3010, Australia; ⊥ Field of Excellence BioHealth, University of Graz, Graz 8010, Austria; # Department of Biochemistry and Pharmacology, University of Melbourne, Parkville, Victoria 3010, Australia; ¶ Bio21 Molecular Science and Biotechnology Institute, University of Melbourne, Parkville, Victoria 3010, Australia

## Abstract

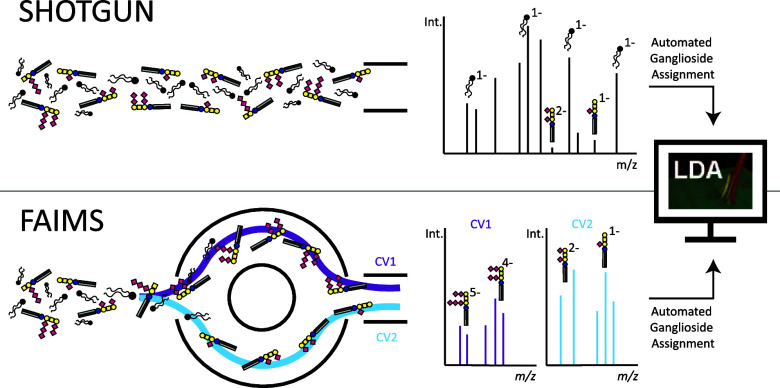

The analysis of gangliosides is extremely challenging, given their structural complexity, lack of reference standards, databases, and software solutions. Here, we introduce a fast 6 min high field asymmetric ion mobility spectrometry (FAIMS) shotgun lipidomics workflow, along with a dedicated software solution for ganglioside detection. By ramping FAIMS compensation voltages, ideal ranges for different ganglioside classes were obtained. FAIMS revealed both class- and charge-state separation behavior based on the glycan headgroup moiety. The number of sialic acids attached to the glycan moiety correlates positively with their preferred charge states, i.e., trisialylated gangliosides were mainly present as [M – 3H]^3–^ ions, whereas [M – 4H]^4–^ and [M – 5H]^5–^ ions were observed for GQ1 and GP1. For data evaluation, we developed a shotgun/FAIMS extension for the open-source Lipid Data Analyzer (LDA), enabling automated annotation of gangliosides up to the molecular lipid species level. This extension utilized combined orthogonal fragmentation spectra from CID, HCD, and 213 nm UVPD ion activation methods and covers 29 ganglioside classes, including acetylated and fucosylated modifications. With our new workflow and software extension 117 unique gangliosides species were identified in porcine brain extracts. While conventional shotgun lipidomics favored the observation of singly charged ganglioside species, the utilization of FAIMS made multiply charged lipid species accessible, resulting in an increased number of detected species, primarily due to an improved signal-to-noise ratio arising from FAIMS charge state filtering. Therefore, this FAIMS-driven workflow, complemented by new software capabilities, offers a promising strategy for complex ganglioside and glycosphingolipid characterization in shotgun lipidomics.

## Introduction

Gangliosides, belonging to the acidic glycosphingolipids, are crucial players in cellular communication and neural function, predominantly present in the brain.^1^ Located on the outer leaflet of the plasma membrane, these molecules contribute to neuronal development, cellular signaling, immune modulation, and show a significant impact on several diseases like cancer, Alzheimer’s disease, and COVID-19.^2,3^ Moreover, gangliosides have emerged as promising marker candidates in stem cell differentiation processes.^4−7^ Structurally, gangliosides feature a lipid moiety (ceramide) and a highly variable glycan headgroup. Sialic acids, as part of the glycan moiety, impart negatively charged character at physiological pH values.^8^ To date, ganglioside analysis remains challenging, given the enormous number of possible structures deriving from the combinations of the diverse and highly variable glycan and lipid moieties. This vast number of feasible structures are covered only to a limited extent by current software tools and databases, along with a limited availability of standards. In the realm of MS-based lipidomics, two predominant methodologies are employed for the analysis: shotgun lipidomics and liquid-chromatography mass spectrometry (LC–MS). Shotgun lipidomics offers the advantages of straightforward sample preparation, rapid analysis, and high-throughput capabilities via the direct infusion of lipid extracts into the instrument.^9^ One major limitation in ganglioside analysis, however, even when using ultrahigh resolution MS, is that signals of low abundance species cannot be distinguished in the presence of high abundance isomers and some isobars, due to the absence of orthogonal separation methods. This reduced specificity is especially pronounced when gangliosides and bulk lipids are analyzed simultaneously from the same sample matrix. Conversely, reversed-phase (RP) LC–MS introduces an additional dimension of separation for complex lipid mixtures.^10^ The specific separation characteristics introduced by LC separation can be exploited by elution-based models such as the equivalent carbon number (ECN) model^11−14^ to distinguish between isobaric or isomeric species, enabling reliable identification of minor lipid species. A drawback of LC–MS lipidomics derives from longer analysis times, which can impede high sample throughput. Shotgun and LC–MS workflows tend to focus on specific ganglioside subsets, resulting in limitations for comprehensive profiling, or depend on long analysis times, and labor-intensive manual annotation.^15−20^ However, due to the biological relevance of these analytes, there is an urgent need for comprehensive and fast deep-profiling approaches. The key for high-throughput and sensitive MS-based glycosphingolipid and ganglioside analyses lies in reducing complexity, i.e., decreasing the number of species that result in a single spectrum. This can be achieved by (1) faster LC–MS methods or (2) introducing an additional dimension of separation orthogonal to *m*/*z*, such as ion mobility spectrometry (IMS).

The general principle underlying IMS is the separation of ions based on their mobility in an electric field, which depends on their size, shape, and charge. The electric field can either be (1) static or (2) oscillating. Furthermore, there are two main separation techniques applied: (1) temporal and (2) spatial separation. The temporal separation gathers information about all ions present at the time of the scan providing information on the arrival time for each ion. The spatial separation acts as a filter, allowing only ions with specific characteristics to pass. By consecutive scanning with different settings a comprehensive view of the ions present can be obtained, as each setting selectively allows specific ions to pass. Several devices with different combinations of the applied electric field and separation technique are commercially available.^21,22^

Numerous studies using IMS based on temporal-separation have been reported for the analysis of gangliosides, offering separation of isomeric ganglioside species and deep characterization of the ganglioside lipidome.^16,17,23−34^ In contrast, spatial-separation based IMS for gangliosides analysis has received limited attention,^35^ despite its potential for increased sensitivity as shown in proteomics and lipidomics studies.^36−44^ Overall, ganglioside analysis approaches aim for high-confidence identifications, requiring accurate mass, MS/MS fragment information, and, ideally, matching database entries or a reference standard.^45^ Collision-induced dissociation (CID) and high-energy collisional dissociation (HCD) are the most commonly used ion activation methods for MS/MS lipid structural analysis. However, ultraviolet photodissociation (UVPD)-MS^n^ represents a cutting-edge technique that provides almost complete structural characterization across multiple lipid categories and classes, including localizing double bond positions, cyclization and other acyl chain modifications, and assignment of *sn*-linkage positions.^39,46−57^

A major bottleneck of ganglioside and glycosphingolipid data analysis is molecular assignment and correct annotation using accurate mass and MS/MS information, which often relies on tedious manual annotation of fragmentation spectra. In 2023, a seminal review provided a comprehensive overview of freely available tools for lipidomics MS-based data analysis,^58^ including tools dedicated for the analysis of shotgun data.^59^ Data evaluation tools for ganglioside based on FAIMS shotgun lipidomics are currently inapplicable for our purpose, because (1) databases entries are limited, (2) higher charge states are completely absent, (3) the available sphingolipid species are exclusively dihydroxylated. Additionally, most tools focus either on shotgun or LC data evaluation and are not explicitly designed for FAIMS. Recently, we demonstrated automated annotation of fragmentation spectra for gangliosides using LC–MS and decision rules.^7^ Here we introduce a newly developed shotgun/FAIMS extension of the freely available software LDA.^60−62^ Importantly, the presented software and the developed decision rules can be applied to both conventional shotgun and FAIMS-based data,^7^ while also supporting UVPD.

In this study, we demonstrate the capability of shotgun MS coupled with spatial-separation-based FAIMS for the enhanced identification of gangliosides. Our investigation further encompasses CID, HCD, and UVPD as fragmentation techniques. We explore the structural insights provided by each fragmentation method and specifically compare the spectral quality between results obtained using shotgun MS approaches, with and without FAIMS. Moreover, we present for the first time an automated solution for annotating ganglioside UVPD data acquired by our FAIMS shotgun approach, which fully exploits the significantly increased sensitivity and flexibility of our method.

## Experimental Section

### Standards and Solvents

All solvents were of LC–MS grade. HPLC grade chloroform (CHCl_3_) was purchased from Ajax Finechem. Butylated hydroxytoluene, isopropanol (IPA), MS grade methanol (MeOH) and acetonitrile (ACN) were purchased from Merck. Ganglioside standards were obtained from Cayman Chemical, Avanti Polar Lipids, and Merck. A pooled ganglioside standard (PGS) including 12 standards with a final concentration of approximately 5 μM was prepared in either (1) 4:2:1 IPA/MeOH/CHCl_3_ (v/v/v) with 20 mM ammonium formate (AF, ChemSupply) or (2) pure 4:2:1 IPA/MeOH/CHCl_3_ (v/v/v), without the addition of AF. Additionally, a total porcine brain extract (BE, Avanti) was chosen as complex proof-of-concept sample. This commercially available sample contains a diverse array of lipid classes, providing a challenging matrix for examining the ability of FAIMS to effectively separate gangliosides from other lipid species. The BE sample was diluted to a final concentration of 250 μg/mL in the two IPA solvent mixtures described above. All standards used in this study are listed in Supporting Information Table 1.

### Mass Spectrometry

In this study, the value of FAIMS for ganglioside analysis has been evaluated in relation to conventional shotgun (referred to as noFAIMS) analysis. Instrument parameters for both setups were optimized, with a focus on spray current, ion transfer tube temperature and the applied radio frequency (RF) voltage (Supporting Information Figure 1). For shotgun analysis, nano electrospray ionization (nESI) using a TriVersa NanoMate (Advion BioSciences, Ithaca, NY, USA) was coupled to an Orbitrap Fusion Lumos mass spectrometer (Thermo Fisher, San Jose, CA, USA). The mass spectrometer (MS) was expanded with a user-installed 213 nm laser (Ekspla NL204, 0.2 mJ, 7–10 ns pulse duration, 1 kHz repetition rate, Vilnius, Lithuania) for UVPD fragmentation. For Full Scan (MS1) experiments, the system was operated in negative ionization mode with a spray voltage of 1.3 kV, and a gas pressure of 0.3 psi. The heated transfer capillary was set to a temperature of 170 °C, the RF voltage was adjusted to 60% and the scan range was set to *m*/*z* 250–2000 using quadrupole isolation and an Orbitrap mass resolving power of 120 000 (at *m*/*z* 200). The automatic gain control (AGC) target was set to standard, and the maximum injection time (MIT) was limited to 100 ms. The scan number was consistently set to 50. Additionally, we developed a 3.5 min automated data-dependent acquisition (DDA) MS/MS method, which includes CID, HCD and UVPD fragmentation for a ganglioside-specific inclusion list (see Supporting Information Figure 2). In detail, 30 s MS1 acquisition was followed by 60 s DDA CID (27%, 10 ms, *Q* = 25), DDA HCD (27%) and DDA UVPD (17 ms) each. These DDA scans were executed in an unscheduled mode using the ganglioside-specific inclusion list with a mass tolerance of 5 ppm and a dynamic exclusion of 10 s. The cycle time was set to 8 s, and an intensity threshold of 8.0 × 10^3^ for the precursor was applied. The Orbitrap mass resolving power was set to 60 000, the *m*/*z* range to 150–2000, with an isolation window of 1.5, 1 microscan, an AGC target of 100% and 300 ms MIT.

For FAIMS measurements, a FAIMS Pro source (Thermo Scientific, Waltham, MA, USA) was used. The spray voltage was set to 1.6 kV, all other parameters were kept constant (see shotgun method above). For MS1, a 2.7 min compensation voltage (CV) scan was designed, starting at a CV of 25 V and reaching 77 V. Each specific CV value was maintained for 6 s before an incremental increase of 2 V in each step. Additionally, a 6 min DDA experiment was conducted for ganglioside identification. Optimized for GD1 gangliosides, the FAIMS CV was fixed at 45 V for 30 s for MS1 scans, followed by CID, HCD and UVDP for 30 s each, with the same settings as described for the noFAIMS setup. From minute 2 to 6, DDA UVPD experiments were performed at different CVs with incremental steps from 35 to 67 V, with each voltage (35, 39, 45, 55, 59, 63, 67 V) held for 30 s.

The PGS and BE samples were measured in triplicates, with both solvent mixtures (i.e., AF and noAF) and both instrument setups (i.e., FAIMS and noFAIMS), along with solvent blanks and QC samples. In-depth explanation of the methodology, along with visual representations and screenshots from the method editing software, can be accessed in the Extended Methods section in the Supporting Information (Section 1: Extended Methods; Tables 2–4 and Figures 2–7). For method validation purposes, we used a ganglioside benchmarking data set for the samples PGS and BE under all four study setups (AF_noFAIMS, noAF_noFAIMS, AF_FAIMS, and noAF_noFAIMS). Based on previously detected gangliosides in standard samples by LC–MS,^7^ the benchmarking list included 112 dihydroxylated ganglioside species from 11 ganglioside classes (Supporting Information ID Table/IDs Benchmarking Data) and was evaluated in Skyline (MacCoss Lab, version 23.1.0.268)^63^ followed by stringent manual curation (present in all 3 replicates, at least 10 data points in MS1, CV range according to the respective class and charge state, presence of diagnostic fragments in MS/MS).

### Development of a New LDA Shotgun/FAIMS Extension

As part of this work, LDA,^62^ which was originally designed for the analysis of chromatography-based MS data, has been extended for the analysis of conventional shotgun and shotgun FAIMS data. This extension comprises an MS1 shotgun quantitation module and a module for isotope correction. For shotgun data, the MS1 quantitation module substitutes LDA’s conventional MS1 quantitation (based on 3D peak deconvolution^60^) by a shotgun specific quantitation based on MS1 intensities. This module extracts for each analyte/ion species combination (*m*/*z* values provided in an extensible Excel mass list) the measured intensities that fall within a user-settable tolerance around the monoisotopic *m*/*z* value of interest (*m* + 0) plus a definable number of isotopologues (*m* + 1, *m* + 2, etc.). For calculating a quantitative surrogate for each isotopologue out of these intensities, three methods are provided, i.e., the “median”, the “mean”, and the “sum”. Subsequently, LDA’s isotope correction routine can be applied. The isotope correction routine works in an iterative manner. It starts with species unaffected by the isotopes of other species, calculates the theoretical values of each isotope (based on the theoretical isotope intensity distribution) of up to *m* + 5, and corrects all the isotopes of other species that show an overlap to the *m*/*z* values of any of these isotopes (from the unaffected species). These theoretical isotope intensities are calculated in relation to the monoisotopic *m* + 0 intensity. The *m* + 0 can be either the measured *m* + 0 intensity, or, if the *m* + 1 has been quantified also, it is the mean of the measured *m* + 0 intensity and a hypothetical *m* + 0 intensity calculated from the measured *m* + 1 intensity. This second calculus may be useful to reduce errors introduced by isotopes of other species, because the *m* + 1 intensity is typically less affected by isotopic overlaps than the monoisotopic *m* + 0 intensity. Then, the algorithm iteratively continues the same procedure with species which reached the “unaffected by other isotopes” status post correction until there are no more species left for correction. Notably, LDA differentiates between species “unaffected” and “affected” by isotopologues originating from other species, and it does not simply start out at species with the lowest *m*/*z* value. This is particularly important for e.g., fully ^13^C labeled species, which tend to show isotopic distributions in negative *m*/*z* direction due to ^12^C impurities. Subsequent to MS1 quantitation, the MS/MS information is evaluated based on decision rule sets.^61^ In a final step, the quantities assessed for the lipid species (sum composition level post isotope correction) are split among the individual lipid molecular species (fatty acid isomers) based on the distinct fragments of each species (for details see ‘Application 3′ of Online Methods of the LDA2 publication^61^).

### Automated Data Evaluation Using LDA

Full MS and DDA scans were analyzed using LDA’s shotgun/FAIMS extension described above (version 2.10.0). The ganglioside specific mass list^7^ was extended to a total of 29 ganglioside classes and modifications (GM1, GM2, GM3, GM4, GM1-Ac, GM1-Fuc, GD1, GD2, GD3, GD1-Ac, GD1-Fuc, GD1-Fuc-Ac, GD3-Ac, GT1, GT2, GT3, GT1-Ac, GT1-Fuc, GT3-Ac, GT3-Fuc, GQ1, GQ1-Ac, GQ1-Fuc, GP1, GH1, GS1, GS1-Fuc, GO1) and the following ion species were searched for: [M – H]^−^ to [M – 8H]^8–^ depending on the respective ganglioside class. An MS1 *m*/*z* tolerance of 5 ppm and a MS/MS precursor tolerance of 10 ppm was applied (these settings are activated in LDA version 2.10.0 by selecting the instrument “OrbiTrap_Lumos_shotgun” and the fragmentation selection ‘Ganglioside_neg_shotgunFAIMS‘). For MS1 quantitation, we obtained the isotope corrected intensities (*m* + 0 and *m* + 1 isotopologues) using the “sum” mode. The evaluation was performed on CID, HCD and UVPD spectra, as well as combined spectra comprising all three ion activation methods, since the combination proved to be most informative for our purpose. Ganglioside (molecular) species annotation and quantitation were performed automatedly by LDA. Only species verified by MS/MS annotations were used for further data processing. Using the rdb export option available in the LDA software, the data was further processed and visualized using R Studio (version 4.3.2) and Illustrator (Adobe Inc., version 28.2, 2023). For R Studio code optimization and refinement, ChatGPT (GPT-3.5, OpenAI, 2023) was used. FreeStyle (Thermo Fisher, 1.8 SP2 QF1, version 1.8.65.0) was used for quality control and visualization along the whole data evaluation process. To validate the performance of the LDA extension, we compared the automated ganglioside annotation list with the manual curated benchmarking list (see Supporting Information ID Table for details).

## Results and Discussion

The structural complexity of gangliosides and glycosphingolipids complicates their analysis using shotgun high-resolution MS-based workflows.

By introducing FAIMS separation prior to shotgun analysis, MS spectra complexity is reduced, offering fast and sensitive ganglioside analysis. Applying FAIMS with combined fragmentation techniques (UVPD, HCD, CID) enabled enhanced hydrophilic interaction liquid chromatography (HILIC)-like separation and automated ganglioside annotation.

### FAIMS for Enhanced Shotgun Ganglioside Detection

To implement shotgun FAIMS for ganglioside detection, fine-tuning of parameters such as sample introduction conditions, ion optics, and spray voltages was essential. Our method optimization demonstrated a substantial enhancement in signal-response by increasing the RF voltage (Supporting Information Figure 1). As no additional in-source fragment formation occurred, general stability of gangliosides within the optimized method was assumed.

#### Evaluation of FAIMS CV Ranges for Different Ganglioside Classes and Charge States

**Figure 1:**
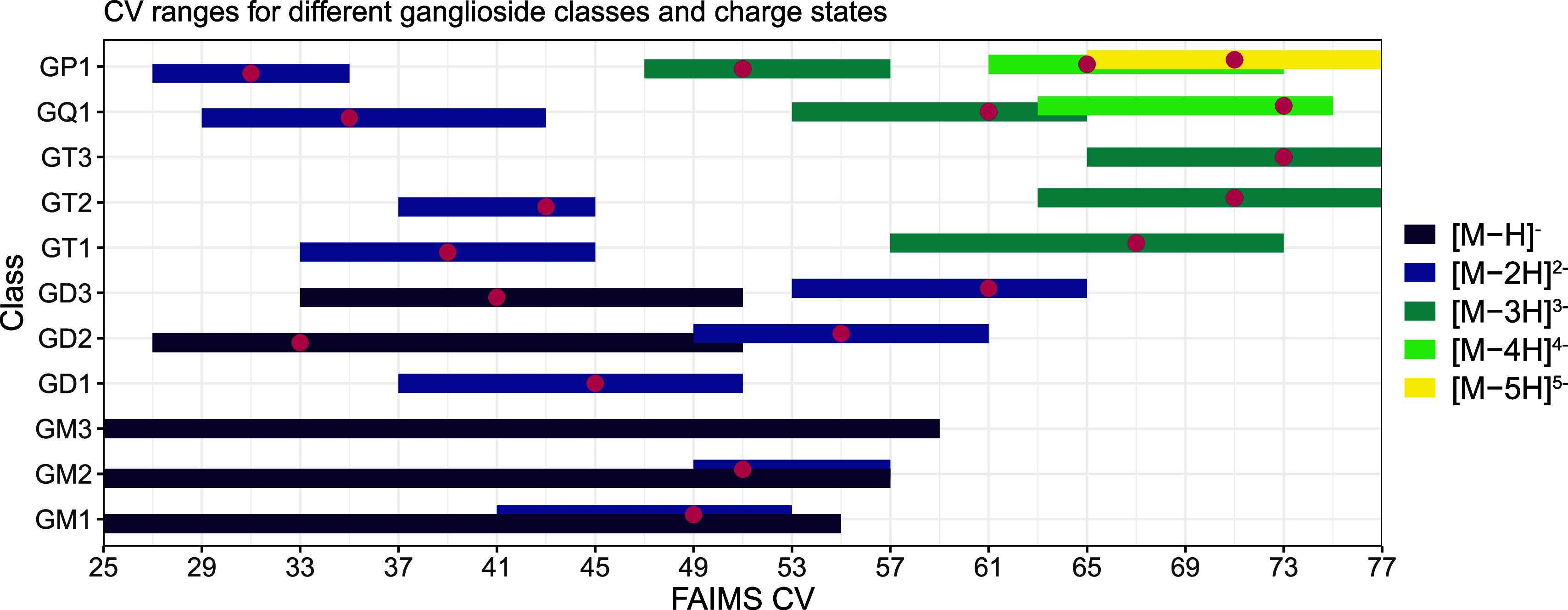
Visualization of CV ranges for each ganglioside class and their corresponding ion species measured in negative ion mode by FAIMS shotgun lipidomics with and without AF. All ganglioside classes can form multiple ion species with different charge states (ranging from [M – H]^−^ to [M – 5H]^5–^, depending on the specific class). The predominant ion species correlates with the number of sialic acids: GM1-3, GD1-3, GT1-3, GQ1 and GP1 species favor the formation of [M – H]^−^, [M – 2H]^2–^, [M – 3H]^3–^, [M – 4H]^4–^, and [M – 5H]^5–^ ions, respectively. Generally, lower CV values promote ions at lower charge states, while higher CV values make multiply charged species accessible. A dependency of ion formation on the presence or absence of AF was observed. The addition of AF promoted the detection of lower-charge state ions, e.g. [M – 2H]^2–^ for GQ1 and GP1 was only observed with the AF containing solvent. Conversely, the absence of AF resulted in the detection of higher-charge state ions, e.g., [M – 4H]^4–^ and [M – 5H]^5–^ for GQ1 or GP1 were only observed in the absence of AF Pink dots indicate the CV_max_, the CV resulting in the highest signal intensity for the respective ganglioside class and charge state. For monosialylated gangliosides no CV_max_ could be determined, as the signal intensity was similar over a broad CV range.

In this study, we explored the CV parameter in FAIMS as an additional separation dimension for shotgun lipidomics analysis. The method validation was performed based on a manually curated benchmarking data set (see Supporting Information ID Table/IDs Benchmarking Data). The FAIMS CV screening method ranging from 25 to 77 V showed that each ganglioside class generally possesses an optimal CV range rather than a narrowly confined voltage requirement. For example, for GD1 [M – 2H]^2–^, a CV range from 37 to 51 V, and for GT1 [M – 3H]^3–^, CV ranges from 57 to 73 V were found to be optimal. Notably, singly charged species were obtained in broader CV ranges as illustrated for GM1, GM2, and GM3 in Figure 1. Additionally, correlation was observed between the number of sialic acids and the most abundant charge state. Singly charged species were dominated by GM1, GM2, and GM3 (single sialic acid) gangliosides, while GD1, GD2, and GD3 (two sialic acids) were observed primarily as doubly charged species.

Following this trend, triply charged species were detected for GT1, GT2, and GT3 (three sialic acids), quadruply charged species for GQ1 (four sialic acids), and even quintuply charged species for GP1 (five sialic acids). Quadruply and especially quintuply charged ion species have only recently been reported for gangliosides.^17,64^ A table with the specific CV ranges for each ganglioside class and their respective charge states can be found in Supporting Information Table 5. Compared to other ion mobility techniques, FAIMS is not capable to provide collisional cross section (CCS) values. However, it is possible to determine the CV maximum (CV_max_), which is the CV corresponding to the highest signal intensity of a respective ganglioside class and charge state by FAIMS. The CV_max_ values are indicated by pink dots in Figure 1 and listed in Supporting Information Table 5. Another interesting observation was that the presence or absence of AF in the solvent mixture influenced the ion species formation. We observed that AF containing samples favored the formation of lower charged species, while higher charged ions were predominantly detected in the absence of AF.

#### FAIMS Provides Class and Charge State Separation

**Figure 2:**
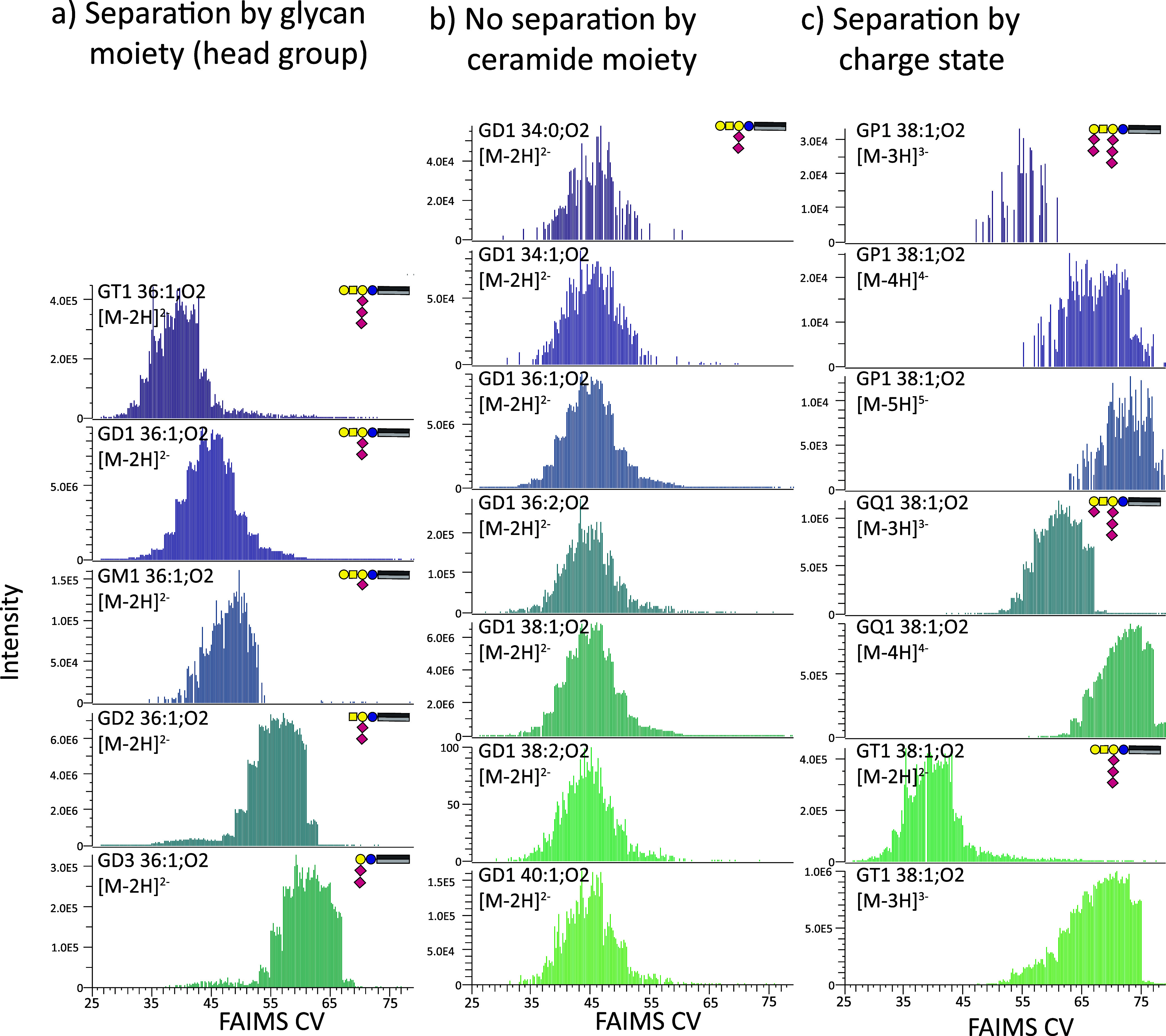
Effect of FAIMS CV range based on (a) the glycan headgroup, (b) ceramide moiety and (c) the charge state. Similar to HILIC separations in LC–MS, FAIMS provides separation based on (a) the headgroup, but (b) not on the ceramide moiety. A higher total sugar length correlates with lower CV ranges, while lower sialic acid numbers lead to higher CV ranges. FAIMS introduces an additional separation dimension, i.e., the charge state (c), with a direct correlation of lower CVs for lower charge states and higher CVs for higher charge states. While both shotgun and LC–MS can discern different charged species, they coelute or they are simultaneously present. Conversely, FAIMS separates differentialy charged species, which is a FAIMS-specific feature. The presented data is acquired from the pooled ganglioside standard analyzed in MS1 without AF in the solvent.

Applying the FAIMS source enabled class-specific separation of gangliosides based on the glycan headgroup (Figure 2a). Two primary factors affect the CV range for a ganglioside class: (1) the total sugar length and (2) the number of sialic acids. A reduction in total sugar length from GD1 to GD2 to GD3 corresponded to an increase in the optimal FAIMS CV (Figures 1 and 2a). Conversely, as illustrated with GT1, GD1, and GM1, an increase in sialic acid content correlated with a lower FAIMS CV range, highlighting the significance of glycan composition in separation dynamics. Notably, all species within a class exhibited the same CV range (Figure 2b), i.e., the total ceramide length and number of double bonds did not influence the CV range for their transmission, as demonstrated with various GD1 lipid species. The headgroup separation observed in FAIMS coupled with the absence of ceramide-based separation offers similar benefits as HILIC in LC–MS. Correspondingly, FAIMS can be seen as a “HILIC-like” separation modality for shotgun lipidomics with an additional layer of specificity to enhance the characterization of complex glycosphingolipid mixtures. State-of-the-art HILIC methods can resolve specific sialic acid positional isomers on the glycan moiety,^20^ which is not possible with FAIMS. However, FAIMS allows fast analysis, as no re-equilibration is required between injections coupled with a straightforward sample preparation, as no ganglioside specific extraction is required. FAIMS further enables the separation of species based on the charge-state, which is unique to FAIMS. Varying CV ranges for distinct charge states are exhibited, with higher charge states requiring higher CV ranges (Figure 2c). As illustrated by the example of 38:1;O2 for the classes GP1, GQ1, and GT1, differently charged ion species are detectable at different CV ranges, following the above-mentioned direct correlation. Notably, the CV range and CV_max_ for a specific class and ion species provided excellent reproducibility, as the values exhibited stability within a sequence and remained consistent in interday comparisons. This reproducibility aligns well with the principles of FAIMS, where the CV is a critical parameter utilized for spatial separation based on variations in electric fields. In summary, spatial-separation based FAIMS, in the context of ganglioside analysis, is primarily influenced by the glycan headgroup (total sugar length and sialic acid quantity) and charge state.

#### FAIMS Improves Signal-to-Noise and Enhances the Spectral Quality

**Figure 3:**
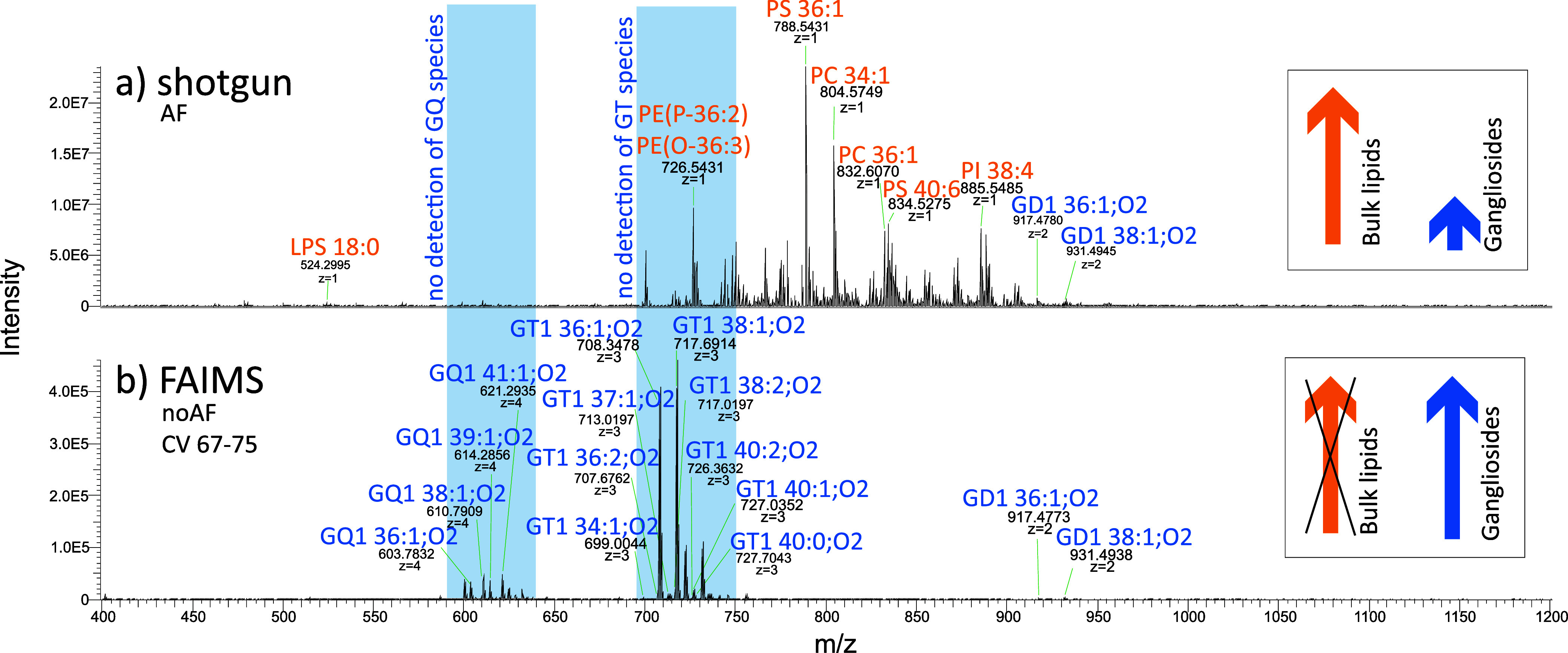
Comparison of ganglioside detection (MS1) in the porcine BE with (a) the conventional shotgun lipidomics workflow using AF as modifier and (b) the FAIMS-based approach without AF. Singly charged bulk lipids can be detected by the conventional shotgun method, but ganglioside species can be hardly observed, particularly species preferring higher charge states. Conversely, FAIMS enables access to the multiply charged ganglioside species. Improvement of the S/N by FAIMS is evident by the detection of the multiply charged GQ and GT species, which were not detected with the conventional shotgun approach.

The impact of FAIMS filtering on both MS1 [signal-to-noise ratio (S/N)] and MS/MS (spectral quality) was examined using a complex porcine BE. While the conventional shotgun (noFAIMS) approach enabled observation of the complete brain lipidome, FAIMS enabled the specific extraction of certain classes and charge states depending on the selected CV (see Figures 1 and 2, as well as Supporting Information Figures 8 and 10). For example, when higher CVs were applied, FAIMS drastically reduced the sample complexity by excluding all singly charged bulk lipids (compare Figure 3a,b). This leads to a significant S/N improvement for ganglioside analysis by FAIMS. FAIMS enabled the detection of highly charged ganglioside species, e.g., several GQs and GTs (Figure 3b), which were not detected by conventional shotgun lipidomics analysis (Figure 3a). As previously reported for peptides,^65,66^ FAIMS filtering demonstrates its applicability for enhanced identification, particularly for enriching highly charged species.

Therefore, we propose to use CV_max_ as an additional identifier to determine gangliosides and other higher charged molecules similar to retention time information used in chromatography-based workflows. This finding is additionally illustrated in Supporting Information Figures 8–10 for GQ1 38:1;O2 and GD1 36:1;O2, where the application of FAIMS resulted in a clean isotopic fine structure for GQ1 38:1;O2 (no overlaps compared to the conventional shotgun approach), and transforms a hybrid MS/MS spectrum into a clean spectrum for GD1 36:1;O2. As the value of a method cannot be fully evaluated by a small set of analytes, we aimed to annotate a broad range of different ganglioside classes and species. To date, the assignment of annotations (MS1 and MS/MS) for gangliosides, and glycosphingolipids in general, is predominantly performed manually, especially in shotgun experiments. We previously introduced an LC-MS-based, automated annotation pipeline for gangliosides utilizing LDA.^7^ In the present study, we extended the LDA for automated annotation of shotgun and FAIMS data of a broad range of ganglioside classes including modified gangliosides as well as various ion activation techniques.

### Enhancing Automated Ganglioside Annotation

A lack of standards, fragment databases, and automated software solutions demands tedious spectra annotation processes, which have to be performed manually to a large extent. To overcome these challenges, we introduce an automated workflow for ganglioside annotation using the newly developed LDA extension, capable of analyzing MS and MS/MS shotgun and FAIMS data.

#### LDA for Automated Shotgun FAIMS Ganglioside Annotation

Whenever a study requires the exploitation of previously undetected ion species, such as the higher negatively charged gangliosides presented in this study, or novel fragmentation techniques like UVPD, automated annotation by conventional software is usually not supported. This is primarily attributed to the absence of informative ions within the databases utilized by these tools. This situation becomes even more challenging when orthogonal separation methods such as FAIMS are used, which are beyond mainstream data analysis. Under these circumstances, flexible and easily expandable solutions become particularly important. The LDA offers a highly adaptable solution by using decision rules,^61^ and target mass lists can be easily extended to any conceivable lipid class and ion species. Besides, LDA supports annotations at the lipid molecular species level, as well as additional hydroxylation states apart from dihydroxylated species,^62^ provides visualization, and extensive export options (Excel, tab delimited text, rdb, mzTab-M,^67^ image formats). To exploit these advantages for shotgun and FAIMS/shotgun data, we developed LDA’s shotgun/FAIMS extension (see Materials and Methods) and tested its usability on pooled ganglioside standards and a porcine BE. The LDA extension is capable of annotating 29 ganglioside classes (including acetylated and fucosylated modifications) and around 8500 distinct ganglioside species.

#### Increased Structural Information by Combining CID, HCD, and UVPD Fragmentation Spectra

**Figure 4:**
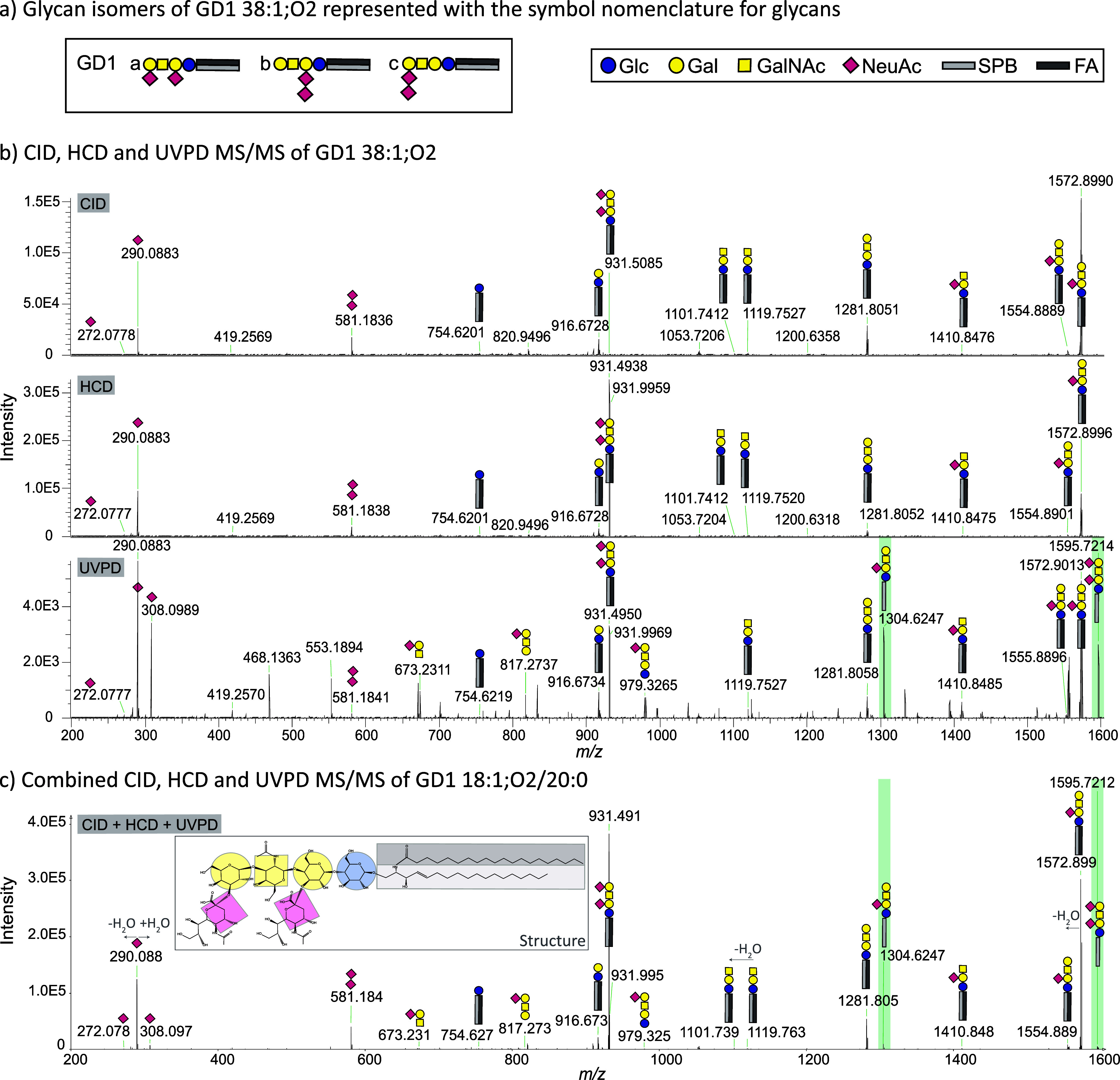
(a) Glycan isomers of GD1 and symbol legend. Glc = glucose, Gal = galactose, GalNAc = *N*-acetylgalactosamine, NeuAc = neuraminic acid/sialic acid, SPB = sphingoid base,^76^ FA = fatty acid. MS/MS (average of 50 scans) for GD1 38:1;O2 in the porcine brain sample using (b) CID, HCD, or UVPD as fragmentation technique. (c) Combined MS/MS spectrum consisting accumulated (sum) of spectra acquired by all three fragmentation techniques. The lipid species level can be determined by all three fragmentation techniques. The UVPD-specific G fragment^46,77^ (neutral loss of FA from precursor) and G-NeuAc (neutral loss of FA and NeuAc from precursor), allows the reliable assignment of the molecular lipid species level (GD1 18:1;O2/20:0) and is highlighted in green. All assignments were performed automatically by the newly developed LDA extension using shotgun FAIMS data without the addition of AF. For visualization, the annotations are represented with the glycan symbol nomenclature.^78,79^

In both shotgun and shotgun FAIMS workflows, we explored CID-, HCD-, and UVPD-MS/MS as distinct fragmentation techniques. This strategy facilitated (1) the comparison of different fragmentation techniques and (2) an increase in the structural information gain by combining MS/MS spectra in an averaged (LDA, Freestyle) or sum (LDA) spectrum, exemplified in Figure 4c. While many fragments could be obtained with all three fragmentation techniques, UVPD resulted in an overall increase in the number of observed fragments. Particularly glycan-specific headgroup fragments were predominantly observed using UVPD (Figure 4b). Nonetheless, the three possible glycan isomers of GD1 remain indistinguishable by shotgun lipidomics (Figure 4a), due to missing separation. In contrast, it is possible to characterize certain isomers on the ceramide moiety, such as resolving the molecular lipid species level as demonstrated in Figure 4c for GD1 38:1;O2 (GD1 18:1;O2/20:0). Theoretically, the molecular lipid species levels could be assigned in some instances using HCD and CID (FA/SPB detection). However, this would often result in false positive annotations, as negative ion mode is insufficient for ceramide characterization using CID and HCD.^7^ With UVPD, characterization of molecular lipid species was possible in several cases (refer to Supporting Information ID Table/IDs Combined Data Overview). This is due to the high reliability of UVPD-generated G fragments^46,77^ (neutral loss of FA from the precursor) or G-NeuAc fragments (neutral loss of FA and sialic acid from the precursor). The high reliability of the G and G-NeuAc fragments was utilized in the LDA extension presented in this study for automated assignment at the molecular species level when these fragments are detected (Figure 4b,c, highlighted in green). Each fragmentation technique generated ganglioside-specific diagnostic fragments, such as *m*/*z* 290.0876, representing sialic acid (NeuAc). Despite expectations based on previously detected double-bond positions in sulfatides and glycerophospholipids,^46,47^ we could not detect double-bond positions or cross-ring (A, X) fragments with UVPD. Their absence may be attributed to the low abundance of ganglioside species in the sample.^47^ Overall, UVPD produces more fragments but leads to fragmentation-rich spectra, which can be an issue for low-abundance species. In such cases, HCD and CID can still detect the necessary diagnostic fragments (e.g., NeuAc, NeuAc–NeuAc), making the combination of all three fragmentation techniques beneficial for ganglioside annotation. An example of the explicit, automatically generated annotation assignment using the LDA extension is provided in Supporting Information Figure 11, demonstrating the detection of GD1-Ac 36:1;O2. An exemplary LDA decision rule is shown in Supporting Information Figure 12.

**Figure 5:**
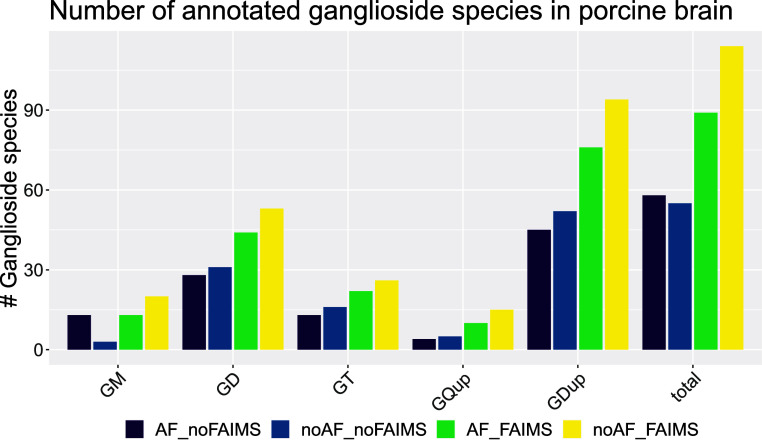
Visualizations of annotations in the porcine BE Comparison of the total number of identifications in the combined data set in respect to the applied method (noFAIMS and FAIMS) in the presence or absence of AF and noAF. And the sialyation degree (GM = monosialylated gangliosides, GD = disialylated gangliosides, GT = trisialylated gangliosides, GQup = quadruply and higher sialylated gangliosides, GDup = di- and higher sialylated gangliosides, total = all classes). For GM species, conventional shotgun lipidomics conditions (AF_noFAIMS) result in similar numbers compared to the FAIMS methods. For all higher sialylated gangliosides FAIMS without AF yielded the highest annotation numbers. In total, the highest identification numbers could be achieved using the FAIMS workflow without AF.

#### Proof-of-Principle: Ganglioside Profiling in Porcine Brain

The utility of FAIMS is particularly evident in the analysis of higher negatively charged gangliosides. The porcine BE serves as an accessible complex biological matrix, predominantly consisting of singly charged bulk lipids (as illustrated in Figure 3a). In the presence of many “noise” lipids, we demonstrate the capabilities of FAIMS in enhancing the detection of gangliosides, as the increased sensitivity and improved spectral quality described above also contribute to higher numbers of ganglioside identifications for the complex porcine brain extract (BE). To validate the performance of the LDA extension, we compared the list of automatically assigned ganglioside annotations (Supporting Information ID Table/IDs LDA unfiltered) to our benchmarking list (Supporting Information ID Table/IDs Benchmarking Data) originating from known ganglioside species. Notably, all MS/MS-confirmed annotations from the benchmarking data set have also been automatically detected by the LDA proving the annotation reliability and general utility of our software extension. While the results generated by LDA appear plausible to us, we cannot completely exclude the possibility of false positives in the presence of isobaric/isomeric species with similar fragmentation patterns as gangliosides. Thus, as for all lipidomics MS data, we recommend the use of commercial standards whenever possible and a manual plausibility verification, particularly if no additional separation method has been applied such as FAIMS or liquid chromatography.

Overall, 153 ganglioside species from 15 ganglioside classes were detected in the pooled gangliosides standard and porcine BE samples using both instrument setups and both solvent mixtures. Including molecular lipid species assignments by adding the MS/MS information on the fatty acid composition (see UVPD specific G fragments in Figure 4d), 250 different gangliosides were detected. This corresponds to 117 unique ganglioside species and 150 IDs including molecular lipid species annotations in the porcine brain. All detected IDs are available in the Supporting Information ID Table/IDs Combined Data Overview. The presented FAIMS assay exploiting UVPD, HCD, and CID at the absence of AF is particularly suitable for GD and other higher sialylated species (Figure 3) leading to 94 ganglioside species annotations with two or more sialic acids (di- and higher sialylated gangliosides) compared to 45 species with conventional shotgun analysis using AF in the BE sample (see Figure 5 and Supporting Information ID Table/IDs Combined Data overview). Overall, FAIMS without AF was superior to the other methods, as demonstrated in Figure 5. FAIMS provides increased coverage of gangliosides as it facilitates the detection of low abundance, higher charge state ganglioside classes. Our findings are in accordance with the literature as GM1, GD1, GD2, GD3, and GT1 have been reported in several studies as the main ganglioside classes present in the human brain.^2,73^ GT3 and GQ1 are less commonly discussed, however, both classes have been described in human brain,^17^ and early ganglioside studies already described GT3 in fish brain,^74^ and GQ1 in rat brain.^75^ The increased number of identifications with FAIMS in a complex biological sample dominated by singly charged molecules highlights the enhancement in sensitivity toward analytes occurring at higher charge states. The advantages of FAIMS in complex samples is further exemplified in the Supporting Information Figure 10, demonstrated by GQ1 38:1;O2. The annotation of this analyte via MS/MS was exclusively achievable using FAIMS emphasizing the general efficacy in detecting and annotating gangliosides. A comprehensive list of all detected ganglioside species in the benchmarking, the LDA-generated and the combined data set, including method details, and information on detected ion species and assigned molecular lipid species in both BE and PGS can be found in the Supporting Information ID Table/IDs Combined Data Details. Ganglioside classes predominantly present as singly charged ions (GM1, GM2, GM3) were promoted by adding AF. As mentioned above, the presence or absence of AF in the solvent mixture influenced the ion species formation. While the stability of the nESI spray remained unaffected by this modification for conventional shotgun analysis, the absence of AF seemingly enhanced the spray stability in FAIMS injections. Furthermore, since the observed charge states depend on AF and the instrument setup, these factors influenced the number of identifications (Figure 5, Supporting Information Tables 6 and 7). This result confirms the advantage of adding AF for conventional shotgun lipidomics analysis,^68^ focusing on singly charged bulk lipids. At the same time, it provides an explanation why most shotgun and LC–MS nontargeted lipidomics workflows primarily detect GM3 or other monosialylated ganglioside classes, while not being capable of detecting higher sialylated ganglioside classes.^69−72^ This underlines the importance of sample preparation and method optimization. Accordingly, salt addition in solvent mixtures should be optimized for the analytes of interest, prior to analysis. Overall, the application of FAIMS in shotgun analyses enables fast and comprehensive ganglioside profiling in samples and complex lipid mixtures. Adding the FAIMS separation dimension increases sensitivity and improves spectral quality. This leads to a higher number of annotations in standard and complex biological samples, which is complemented by LDA’s automated ganglioside annotation solution.

## Conclusion

In this work, we introduce a new shotgun FAIMS assay for ganglioside analysis complemented by an automated annotation solution. FAIMS shotgun lipidomics offers rapid class- and charge-state separation of gangliosides, providing increased sensitivity for multiply charged ganglioside species in the presence of bulk lipids. The FAIMS workflow, coupled with extended structural information obtainable by a combination of CID, HCD, and UVPD significantly enhances the annotation of gangliosides in pooled standards and porcine brain samples. Conventional shotgun lipidomics favors singly charged gangliosides and bulk lipids, while FAIMS excels in detecting multiply charged species, particularly higher sialylated gangliosides. If simultaneous bulk lipid analysis is required, it is advisable to conduct conventional shotgun analysis, followed by shotgun FAIMS analyses for gangliosides or other multiply charged analytes. Although the addition of AF is beneficial for singly charged lipid species with the shotgun workflow, it is counterproductive for gangliosides or higher-charged lipids in the FAIMS workflow.

In summary, this study provides, for the first time, a clear strategy for reproducibly exploiting FAIMS filtering based on spatial separation in an oscillating electrical field for unambiguous ganglioside analyses. FAIMS is not capable of a CCS readout but offers the introduced concept of class and charge state ganglioside separation using CV ranges. The CV_max_ can serve as an additional identification parameter similar to the ECN model in LC–MS. Integrating FAIMS with LC–MS appears feasible with scan time optimization and appropriate flow regimes. As FAIMS offers HILIC-like separations, pairing it with RP-MS could yield highly orthogonal chemical information. While HILIC is able to resolve certain sialic acid positional isomers using a minimum runtime of 15 min,^20,80−82^ shotgun FAIMS provides a faster (6 min) ganglioside profiling strategy with class and charge-state separation and straightforward sample preparation.

The presented FAIMS shotgun workflow is complemented by a software solution for fully automated annotation of 29 classes corresponding to around 8500 distinct gangliosides species. We anticipate that our work can be easily utilized by anyone interested in in-depth analysis of gangliosides and will support automated annotation approaches. The flexibility of extending the LDA for new ganglioside classes/modifications and additional fragmentation techniques is particularly interesting for researchers working on new lipid classes. Overall, FAIMS shotgun lipidomics, especially when coupled with advanced MS/MS methods, is a promising tool for the enhanced separation and structural annotation of multiply charged molecules, such as glycosphingolipids.

## Supporting Information Available

Extended Experimental section and supporting tables and figures (PDF)
Supporting Information ID Table (XLSX)
Installation and download instruction for LDA 2.10.0 (PDF)
Access to study data (PDF)
