# Supplementary material for: FAIMS Shotgun Lipidomics for Enhanced Class- and Charge-State Separation Complemented by Automated Ganglioside Annotation
Source: Anal Chem. 2024 Jul 19;96(30):12296–307. doi: 10.1021/acs.analchem.4c01313 (PMC11295132; doi:10.1021/acs.analchem.4c01313)
Supplement: Supplementary file 1 — ac4c01313_si_001.pdf [file ac4c01313_si_001.pdf]

# FAIMS Shotgun Lipidomics for Enhanced Class- and Charge-State Separation Complemented by Automated Ganglioside Annotation

Katharina Hohenwallner[1,2], Leonida M. Lamp[3], Liuyu Peng[4], Madison Nuske[4], Jürgen Hartler[3,5*], Gavin E. Reid[4,6,7*], Evelyn Rampler[1,2*]

1 Department of Analytical Chemistry, Faculty of Chemistry, University of Vienna, 1090 Vienna, Austria.
2 Vienna Doctoral School in Chemistry (DoSChem), University of Vienna, 1090 Vienna, Austria.
3 Institute of Pharmaceutical Sciences, University of Graz, 8010 Graz, Austria.
4 School of Chemistry, University of Melbourne, Parkville, Victoria 3010, Australia.
5 Field of Excellence BioHealth, University of Graz, 8010 Graz, Austria.
6 Department of Biochemistry and Pharmacology, University of Melbourne, Parkville, Victoria 3010, Australia.
7 Bio21 Molecular Science and Biotechnology Institute, University of Melbourne, Parkville, Victoria 3010, Australia.

**Contents**

1 Extended Methods ........ 2
1.1 Standards and solvents ........ 2
1.1.1 Overview of the final experimental setup ........ 2
1.2 Method development: Examining the influence of RF voltage on signal response ........ 2
1.2.1 Method development considerations: Cycle time and fragmentation techniques ........ 3
1.3 Shotgun (no FAIMS) methods ........ 4
1.4 Shotgun FAIMS methods ........ 6
2 Extension: Evaluation of FAIMS CV ranges for different ganglioside classes and charge states ........ 8
3 Extension: FAIMS improves signal to noise and enhances the spectral quality ........ 9
4 Extension: Enhancing automated ganglioside annotation with the LDA ........ 12

# 1 Extended Methods

## 1.1 Standards and solvents

A list of all used standards including the catalogue number can be found in the Supplementary Table 1. A pooled ganglioside standard (PGS), including standards 1-11 from the Supplementary Table 1, was diluted to a final concentration of approximately 5 µM (calculated for the species 36:1;O2 of the respective standard mixture).

**Supplementary Table 1.** Standards used in the present study and corresponding manufacturer and catalogue number.

| No | Sample Name | Manufacturer | Catalogue No. |
|---|---|---|---|
| 1 | GD1a | Avanti | 860055P |
| 2 | GD1b | Avanti | 860056P |
| 3 | GD2 | Cayman | 25487 |
| 4 | GD3 | Merck | 345752 |
| 5 | GM1 | Cayman | 19579 |
| 6 | GM3 | Avanti | 860058P |
| 7 | GQ1b | Merck | 345754 |
| 8 | Lactosylceramide and Ganglioside GM3 and GD3 Mixture | Cayman | 29361 |
| 9 | Neutral Glycosphingolipid Mixture | Cayman | 29360 |
| 10 | Disialoganglioside Mixture | Cayman | 29358 |
| 11 | Total Ganglioside Extract (TGE) | Avanti | 860053P |
| 12 | Porcine total brain extract (BE) | Avanti | 131101C |

### 1.1.1 Overview of the final experimental setup

Supplementary Table 2 provides an overview of the different experimental shotgun and shotgun FAIMS setups using 4/2/1 (v/v/v) IPA/MeOH/$CHCl_3$ as solvent at the presence and absence or ammonium formate (AF) as modifier. For both setups MS1 and DDA MS/MS data were acquired, the run times for each method are indicated. Several samples were measured, including a porcine brain extract (BE), a pooled ganglioside standard (PGS) as well as solvent blanks and quality control samples, which were injected multiple times along the sequence. The pooled ganglioside standard (PGS) and porcine brain extract (BE) (see Supplementary Table 1 and 2) were measured in triplicate, with both solvent mixtures (i.e., AF and noAF) and both instrument setups (i.e., FAIMS and noFAIMS), along with solvent blanks and QC samples.

**Supplementary Table 2**. Overview of the conducted experiments for shotgun with and without FAIMS. BE=porcine brain extract; PGS=pooled ganglioside standard.

| Setup | Solvent | Sample | MS |
|---|---|---|---|
| No FAIMS | 421 + AF<br>421 no AF | BE (250 µg/mL)<br>PGS (5 µM)<br>QC (BE)<br>Solvent Blanks | MS1: 50 scans, 40 sec<br>MS/MS: auto scan (HCD, CID, UVPD), 3.5 min |
| FAIMS | 421 + AF<br>421 no AF | BE (250 µg/mL)<br>PGS (5 µM)<br>QC (BE)<br>Solvent Blanks | MS1: 1) 50 scans, CV 45, 40 sec<br>2) CV scan, CV 25-77, 2V steps, 2.7 min<br>MS/MS: auto scan (HCD, CID, UVPD), 6 min |

## 1.2 Method development: Examining the influence of RF voltage on signal response

Ion optics are among the most influential factors in MS-based ganglioside analysis, affecting signal response and in-source fragmentation. In our study, we systematically investigated the impact of radio frequency (RF) voltages ranging from 5% to 80% (with a 1% increment and 10 seconds per voltage), on gangliosides. The results revealed a substantial improvement of approximately one order of magnitude in signal response as RF voltages increased. Gangliosides are relatively stable analytes compared to some other lipid classes. They typically exhibit detectable sialic acid fragments (*m/z* 290.0881) in negative ionization mode. Despite the drastic improvement in signal intensity for the intact analyte precursor ions with increasing RF voltage, no sialic acid fragments were observed in Full MS spectra. This suggests the absence of any significant in-source fragmentation for the respective ganglioside. Notably, the most pronounced increase in signal response occurred between 5% and 60%, with marginal improvements from 60% to 80%. Thus, we adopted 60% as the optimal RF voltage for our final method. The ion transfer tube temperature (150 °C and 170 °C) and ionization voltage settings (ranging from 1.1 kV to 1.7 kV) exhibited a comparatively modest influence on signal response in the tested configurations. A temperature of 170 °C yielded a slightly enhanced signal response, consequently it was used for the final setup. For the shotgun lipidomics (noFAIMS) setup, an optimal spray voltage of 1.3 kV was determined. In contrast, for the FAIMS configuration, a higher voltage of 1.6 kV demonstrated the best results in terms of signal response.

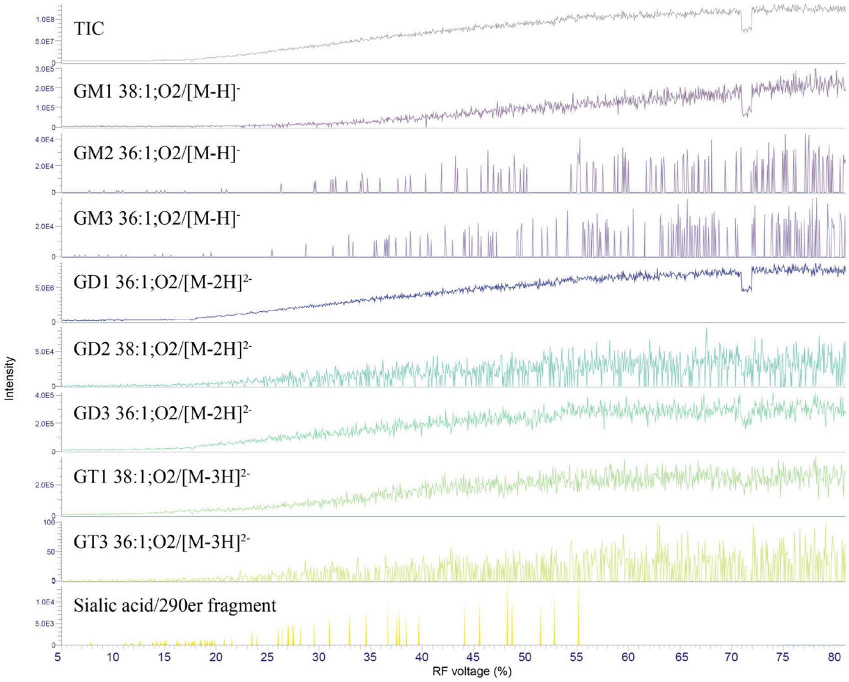


**Supplementary Figure 1.** Impact of increasing RF voltages on signal intensity. EICs of several ganglioside species from different classes have been extracted, as well as the m/z 290.0881 (sialic acid fragment), which usually represents the most labile bond within gangliosides. It can be seen that there is no significant increase in the intensity of the sialic acid fragment. At the same time, the signal intensity of the corresponding species increases with higher RF voltages. It can be concluded that using higher RF voltages is beneficial for the ionization of gangliosides promoting in-source fragmentation.

### 1.2.1 Method development considerations: Cycle time and fragmentation techniques

The cycle time in high-resolution tandem mass spectrometry (HR-MS/MS), particularly in systems like the Orbitrap, refers to the total time required to complete one full sequence of MS1 and MS/MS measurements before restarting the sequence. This cycle includes the MS1 scan (precursor ion detection), fragmentation, and the MS/MS scan (product ion detection). For Orbitrap instruments, the cycle time heavily depends on the resolution setting. Higher resolution settings provide more detailed and accurate mass measurements but require more time for each scan. Consequently, optimizing cycle time involves balancing the desired resolution with maintaining a sufficient speed to analyze complex samples efficiently. In our shotgun and shotgun FAIMS methods we defined several sub-experiments, including 30 seconds MS1 acquisition only, followed by DDA experiments using CID, HCD or UVPD as fragmentation technique. For the DDA experiments the cycle time was set to 8 seconds (detailed explanations are available in 1.3 and 1.4).. For shotgun analyses, it is advisable to generate several scans, which are usually averaged during data analysis. In traditional shotgun experiments using the nanoMate, the limiting factor is the spray stability with the given injection volumes. In our study, we used 8 µL injections, resulting in more than 15 minutes of stable sprays. We aimed for a rapid ganglioside shotgun screening while being able to comprehensively compare HCD, CID, and UVPD fragmentation techniques for ganglioside annotation, as each fragmentation technique offers unique advantages:

- UVPD: This technique generates characteristic fragments for reliable annotation at the lipid molecular species level in negative ion mode (G fragment). Using HCD or CID, such annotations can be reliably achieved in positive ion mode only, however, the ionization efficiency for gangliosides is much worse in positive ion mode. In the updated LDA version, we utilize the G fragment for molecular lipid species assignment of gangliosides (see Supplementary Information ID Table). However, UVPD can produce more fragments, leading to fragmentation-rich spectra, which can be problematic for low-abundance species.
- HCD and CID: These are state-of-the-art fragmentation techniques essential for detecting necessary and well-known diagnostic fragments (e.g., NeuAc 290 or NeuAc-NeuAc 581). While fragment-rich UVPD spectra allow for a more detailed structural characterization, their ion yield is lower than for HCD and CID. Therefore, for species at low abundance, the supplementation of HCD and CID spectra allows at least for confirmation at the lipid species level.

The combination of state-of-the-art HCD, CID fragmentation, and UVPD producing fragmentation-rich spectra provides comprehensive ganglioside analysis and entails richer MS/MS data to increase both identification coverage and improved structural characterization. The respective methods are described in more detail in section 1.3 and 1.4 below.

### 1.3 Shotgun (no FAIMS) methods

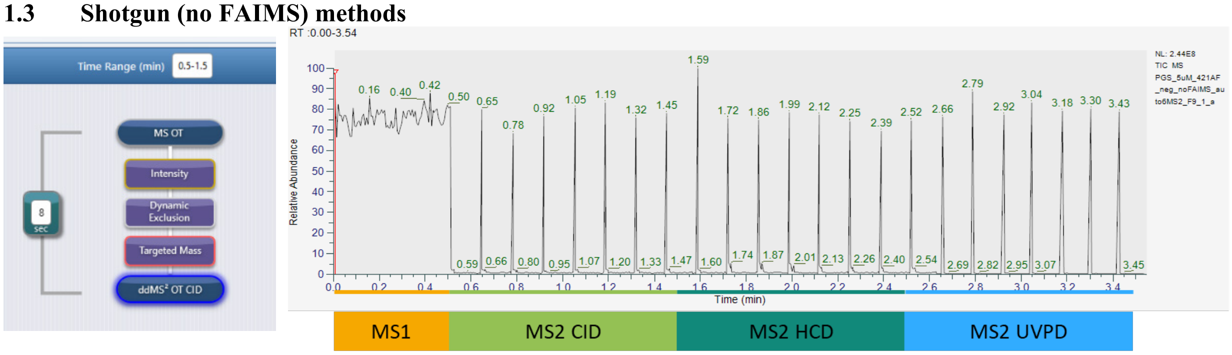


**Supplementary Figure 2**. Overview of the DDA method for shotgun (no FAIMS) using CID, HCD and UVPD fragmentation.

For shotgun analysis, nano electrospray ionization (nESI) using a TriVersa NanoMate (Advion BioSciences, Ithaca, NY, USA) was coupled to an Orbitrap Fusion Lumos mass spectrometer (Thermo Fisher, San Jose, CA, USA). The mass spectrometer (MS) was expanded with a user-installed 213 nm laser (Ekspla NL204, 0.2 mJ, 7-10 ns pulse duration, 1 kHz repetition rate, Vilnius, Lithuania) for UVPD fragmentation. For the Full Scan (MS1), the system was operated in negative ionization mode with a spray voltage of 1.3 kV, and a gas pressure of 0.3 psi. The heated transfer capillary was set to a temperature of 170 °C, the radio frequency (RF) voltage was adjusted to 60% and the scan range was set to *m/z* 250-2,000 using quadrupole isolation and an Orbitrap mass resolving power of 120,000 (at *m/z* 200). The Automatic Gain Control (AGC) target was set to standard, and the maximum injection time (MIT) was limited to 100 ms. The scan number was consistently set to 50. Additionally, we developed a 3.5-minutes automated data-dependent acquisition (DDA) MS/MS method, which includes CID, HCD and UVPD fragmentation for a ganglioside-specific inclusion list (see Supplementary Figure 2). In detail, 30 seconds MS1 acquisition was followed by 60 seconds DDA CID (27%, 10 ms, Q=25), DDA HCD (27%) and DDA UVPD (17 ms) each. These DDA scans were executed in an unscheduled mode using the ganglioside-specific inclusion list with a mass tolerance of 5 ppm and a dynamic exclusion of 10 seconds. The cycle time was set to 8 seconds, and an intensity threshold of $8.0 \times 10^3$ for the precursor was applied. The Orbitrap mass resolving power was set to 60,000, the m/z range to 150-2,000, with an isolation window of 1.5, 1 microscan, an AGC target of 100% and 300 ms MIT. An overview of the set parameters can be found in the Supplementary Table 3 and Supplementary Figure 3.

The 3.5-minute shotgun method was set up to perform 30 seconds of MS1 scans, followed by 1 minute of autoMS/MS (using an inclusion list) for each of HCD, CID, and UVPD, resulting in a total run time of 3.5 minutes. This means an additional one minute per fragmentation technique, which remains significantly faster than traditional HILIC measurements (usually >12 minutes) and not much longer than traditional shotgun measurements. At the same time, this approach enriches the MS/MS information exploiting different fragmentation mechanisms.

**Supplementary Table 3.** MS parameters for the automated MS/MS method for shotgun (no FAIMS).

| Parameter (MS/MS) | CID | HCD | UVPD |
|---|---|---|---|
| Default Charge state | 2 | 2 | 2 |
| Cycle Time | 8 sec | 8 sec | 8 sec |
| Intensity Threshold | $8.0 \times 10^3$ | $8.0 \times 10^3$ | $8.0 \times 10^3$ |
| Dynamic exclusion | After 5 times for 30 sec | After 5 times for 30 sec | After 5 times for 30 sec |
| Inclusion list | Yes | Yes | Yes |
| Isolation window (*m/z*) | 1.5 | 1.5 | 1.5 |
| Collision Energy | 27% (fixed, 10 ms, Q=0.25) | 27% (fixed, normalized) | 17 ms excitation time |
| Resolution | 60,000 | 60,000 | 60,000 |
| Scan Range | 150-2000 | 150-2000 | 150-2000 |
| Normalized AGC | Standard (100%) | Standard (100%) | Standard (100%) |
| MIT | 300 | 300 | 500 |

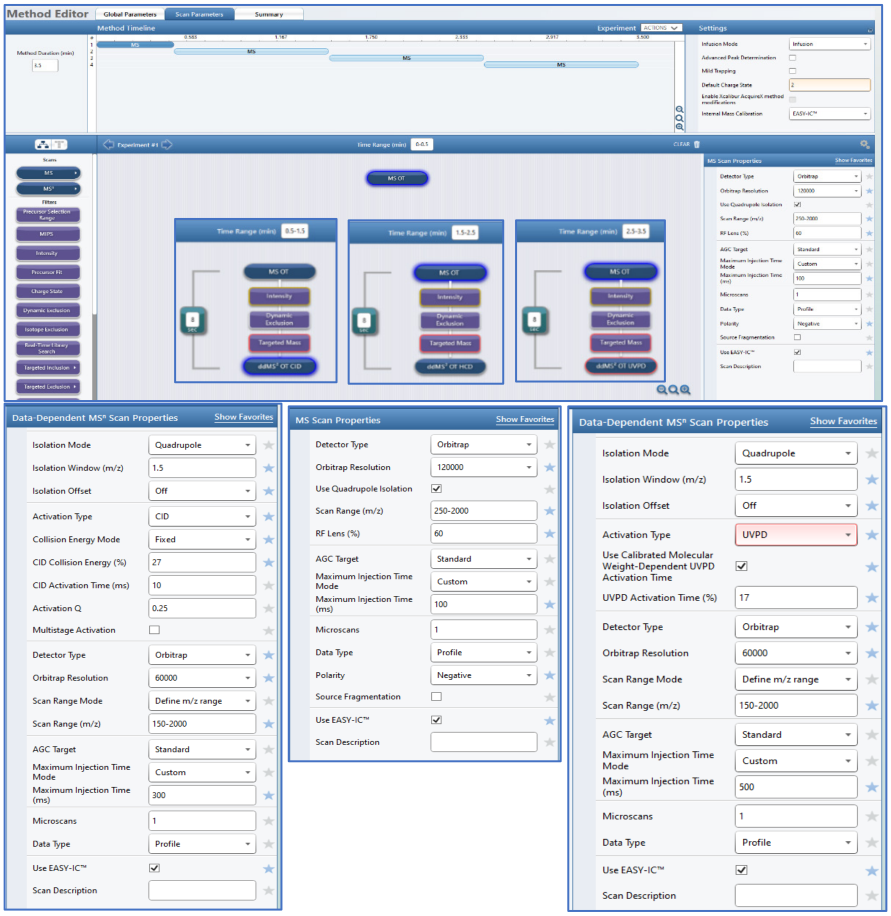


**Supplementary Figure 3.** Screenshot of the Method Editor (Thermo) with detailed settings for the shotgun MS/MS (DDA) method.

## 1.4 Shotgun FAIMS methods

For FAIMS measurements, a FAIMS Pro source (Thermo Scientific, Waltham, MA, USA) was used. The spray voltage was set to 1.6 kV, all other parameters were kept constant (see shotgun method in section 1.3 above). For MS1, a 2.7 min compensation voltage (CV) scan was designed, starting at a CV of 25 V and reaching 77 V. Each specific CV value was maintained for 6 seconds before an incremental increase of 2 V in each step (see Supplementary Figure 4 and 5).

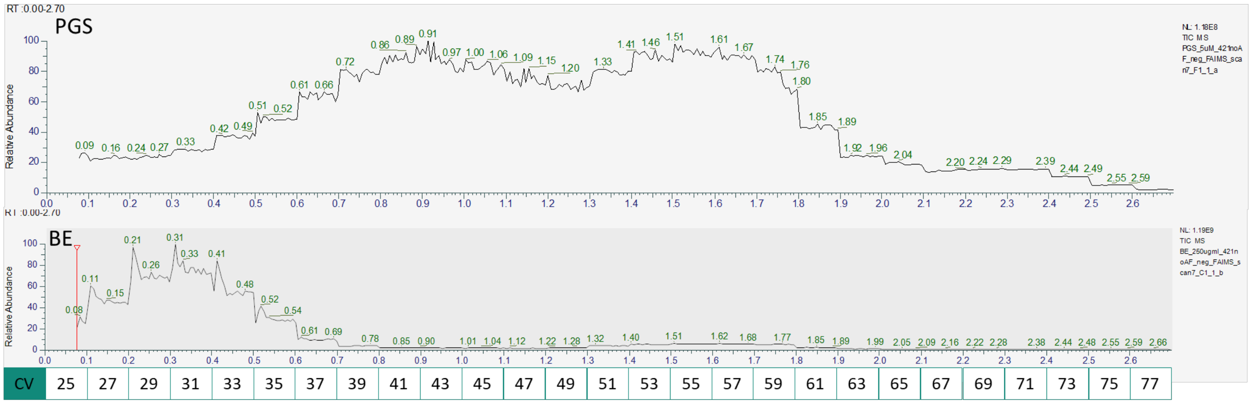


**Supplementary Figure 4**. Visualization based on the TIC of the pooled ganglioside standard (PGS, top) and brain extract (BE, bottom) of the FAIMS CV screening method (MS1), increasing CV steps from 25 to 77 CV are shown.

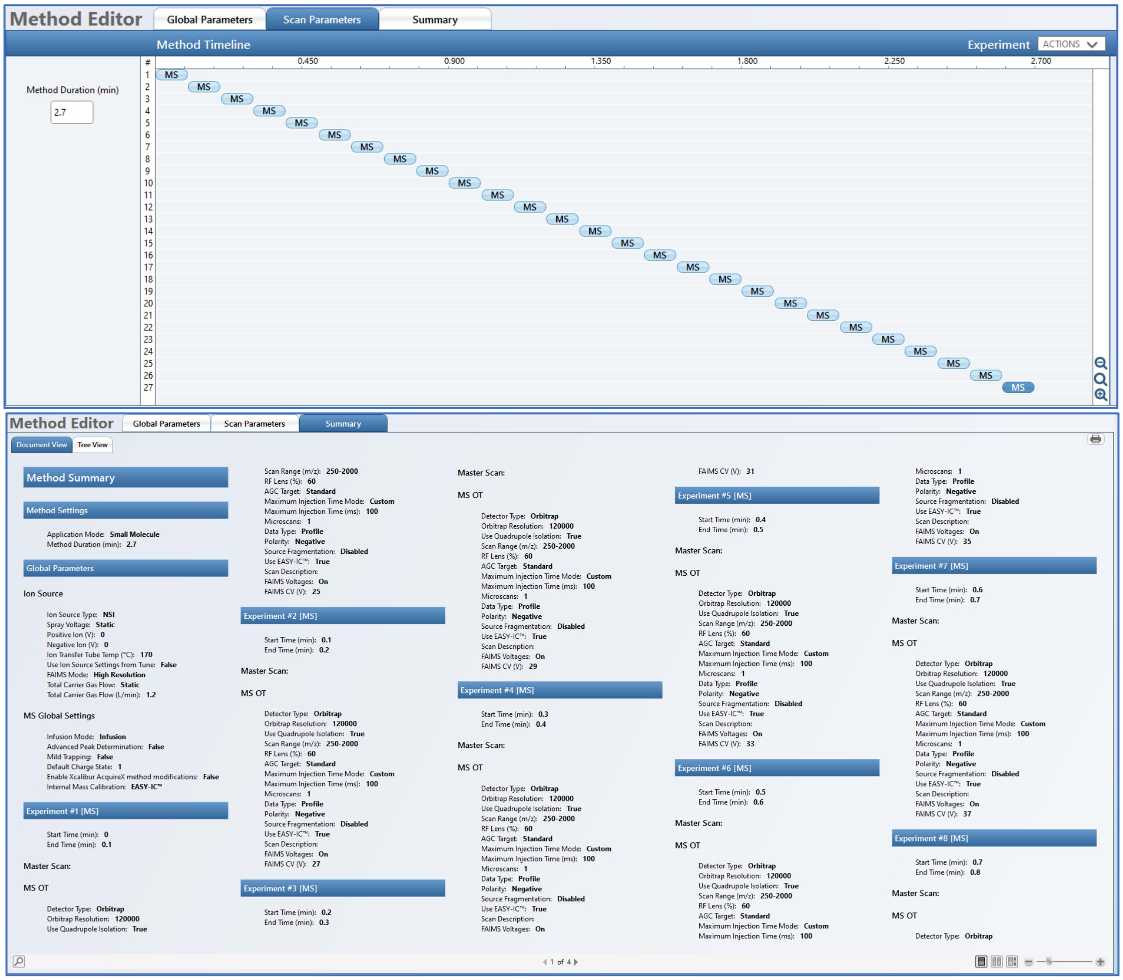


**Supplementary Figure 5.** Method Editor screenshot with detailed settings for the FAIMS CV scan (MS1).

Additionally, a 6-minutes MS/MS (DDA) experiment was conducted for ganglioside identification. Optimized for GD1 gangliosides, the FAIMS CV was fixed at 45 V for 30 seconds for MS1 scans, followed by DDA CID, HCD and UVDP for 30 seconds each, with the same settings as described for the noFAIMS setup. From minute 2 to 6, DDA UVPD experiments were performed at different CVs with incremental steps from 35 V to 67 V, with each voltage (35 V, 39 V, 45 V, 55 V, 59 V, 63 V, 67 V) held for 30 seconds. See Supplementary Figure 6 for visualization and Supplementary Figure 7 and Supplementary Table 4 for detailed parameters.

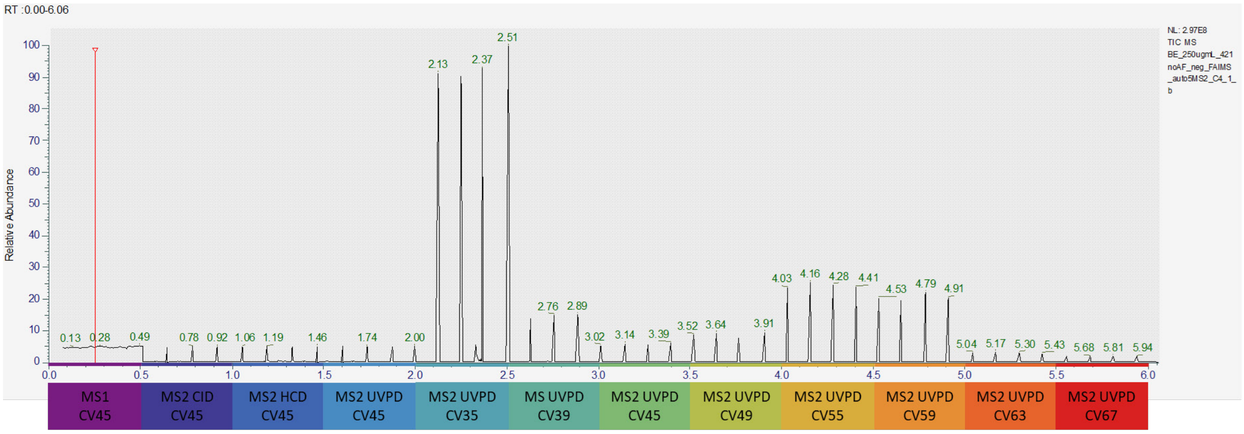


**Supplementary Figure 6.** Schematic overview of automated DDA shotgun FAIMS method with corresponding CVs and the respective fragmentation techniques.

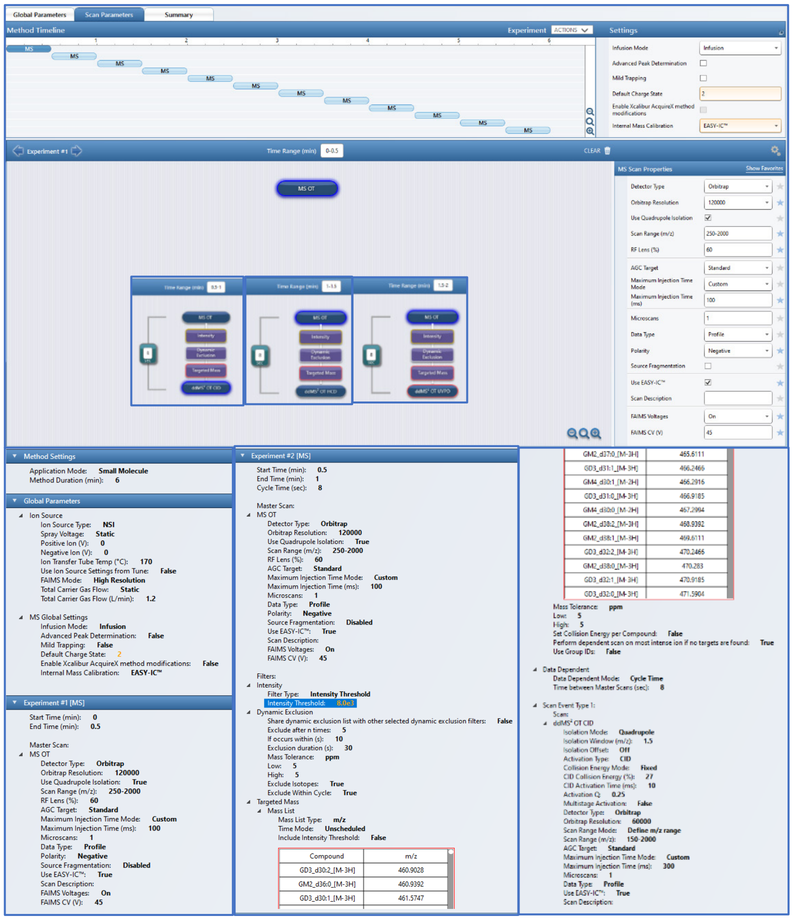

**Supplementary Figure 7.** Screenshots of the Method Editor showing detailed settings of the FAIMS MS/MS (DDA) method.

**Supplementary Table 4**. Detailed overview of FAIMS MS/MS (DDA) method including CID, HCD and UVPD fragmentation. The sequence of the applied techniques including their time ranges are shown.

| Time [min] | Scan mode | CV | Fragmentation |
|---|---|---|---|
| 0.0-0.5 | MS1 | 45 | *NA* |
| 0.5-1.0 | DDA MS/MS | 45 | CID |
| 1.0-1.5 | DDA MS/MS | 45 | HCD |
| 1.5-2.0 | DDA MS/MS | 45 | UVPD |
| 2.0-2.5 | DDA MS/MS | 35 | UVPD |
| 2.5-3.0 | DDA MS/MS | 39 | UVPD |
| 3.0-3.5 | DDA MS/MS | 45 | UVPD |
| 3.5-4.0 | DDA MS/MS | 49 | UVPD |
| 4.0-4.5 | DDA MS/MS | 55 | UVPD |
| 4.5-5.0 | DDA MS/MS | 59 | UVPD |
| 5.0-5.5 | DDA MS/MS | 63 | UVPD |
| 5.5-6.0 | DDA MS/MS | 69 | UVPD |

## 2 Extension: Evaluation of FAIMS CV ranges for different ganglioside classes and charge states

Optimal CV ranges were determined for 11 ganglioside classes, showing a correlation between sialic acid content and charge states. Notably, singly charged species exhibited broader CV ranges. The presence of ammonium formate in the solvent influenced ion species formation, favoring lower charged species with AF and higher charged ions without AF. FAIMS provided class-specific separation based on the glycan head group, with CV ranges influenced by total sugar length and sialic acid quantity. Unlike the ceramide structure, the glycan head group was a critical determinant in FAIMS separation within glycosphingolipid classes. FAIMS, similar to HILIC in LC-MS, offered class specific separations based on the head group with an additional layer of specificity for charge states. In the Supplementary Table 5, the determined FAIMS CV ranges for each ganglioside class and ion species are summarized. Additionally, the $CV_{max}$ (compensation voltage yielding the highest signal intensity for a respective ganglioside class and charge state) is documented. It has to be mentioned that for monosialylated ganglioside no clear $CV_{max}$ for the ion species $[M-H]^-$ could be determined. The singly negatively charged GM classes have been present over a broad CV range. It is possible that their $CV_{max}$ is below the investigated CVs in this study (CV 25-77).

**Supplementary Table 5.** Overview of ion species specific CV ranges and determined $CV_{max}$ for different ganglioside classes and charge states. The predominant ion species are highlighted in bold.

| Ganglioside class | CV $[M-H]^-$ | CV $[M-2H]^{2-}$ | CV $[M-3H]^{3-}$ | CV $[M-4H]^{4-}$ | CV $[M-5H]^{5-}$ |
|---|---|---|---|---|---|
| GM1 | **25-55** | 41-53 | | | |
| GM2 | **25-57** | 49-57 | | | |
| GM3 | **25-59** | | | | |
| GD1 | | **37-51** | | | |
| GD2 | 27-51 | **49-61** | | | |
| GD3 | 33-51 | **53-65** | | | |
| GT1 | | 33-45 | **57-73** | | |
| GT2 | | **37-45** | **63-77** | | |
| GT3 | | | **65-77** | | |
| GQ1 | | 29-43 | **53-65** | **63-75** | |
| GP1 | | 27-35 | 47-57 | **61-73** | **67-77** |
| Ganglioside class | $CV_{max}$ $[M-H]^-$ | $CV_{max}$ $[M-2H]^{2-}$ | $CV_{ma}$x $[M-3H]^{3-}$ | $CV_{max}$ $[M-4H]^{4-}$ | $CV_{max}$ $[M-5H]^{5-}$ |
| GM1 | | 49 | | | |
| GM2 | | 51 | | | |
| GM3 | | | | | |
| GD1 | | **45** | | | |
| GD2 | 33 | **55** | | | |
| GD3 | 41 | **61** | | | |
| GT1 | | 39 | **67** | | |
| GT2 | | **43** | **71** | | |
| GT3 | | | **73** | | |
| GQ1 | | 35 | **61** | **73** | |
| GP1 | | 31 | 51 | **65** | **71** |

## 3 Extension: FAIMS improves signal to noise and enhances the spectral quality

FAIMS significantly enhances signal-to-noise (S/N) and spectral quality in comparison to conventional shotgun approaches. Investigating its impact on MS1 and MS/MS levels using porcine brain extract, FAIMS acts as a filter, selectively extracting specific lipid classes and charge states based on the chosen compensation voltage (CV). While conventional shotgun allows observation of the complete brain lipidome, FAIMS reduces sample complexity, leading to substantial S/N improvements for ganglioside analysis. FAIMS enables the detection of highly charged ganglioside species that are missed in traditional shotgun lipidomics (see Supplementary Figure 8a). The electric field-based separation by FAIMS eliminates isobaric overlaps, illustrated for GQ1 38:1;O2 in Supplementary Figure 8c and GD1 36:1;O2 in Supplementary Figure 9b.

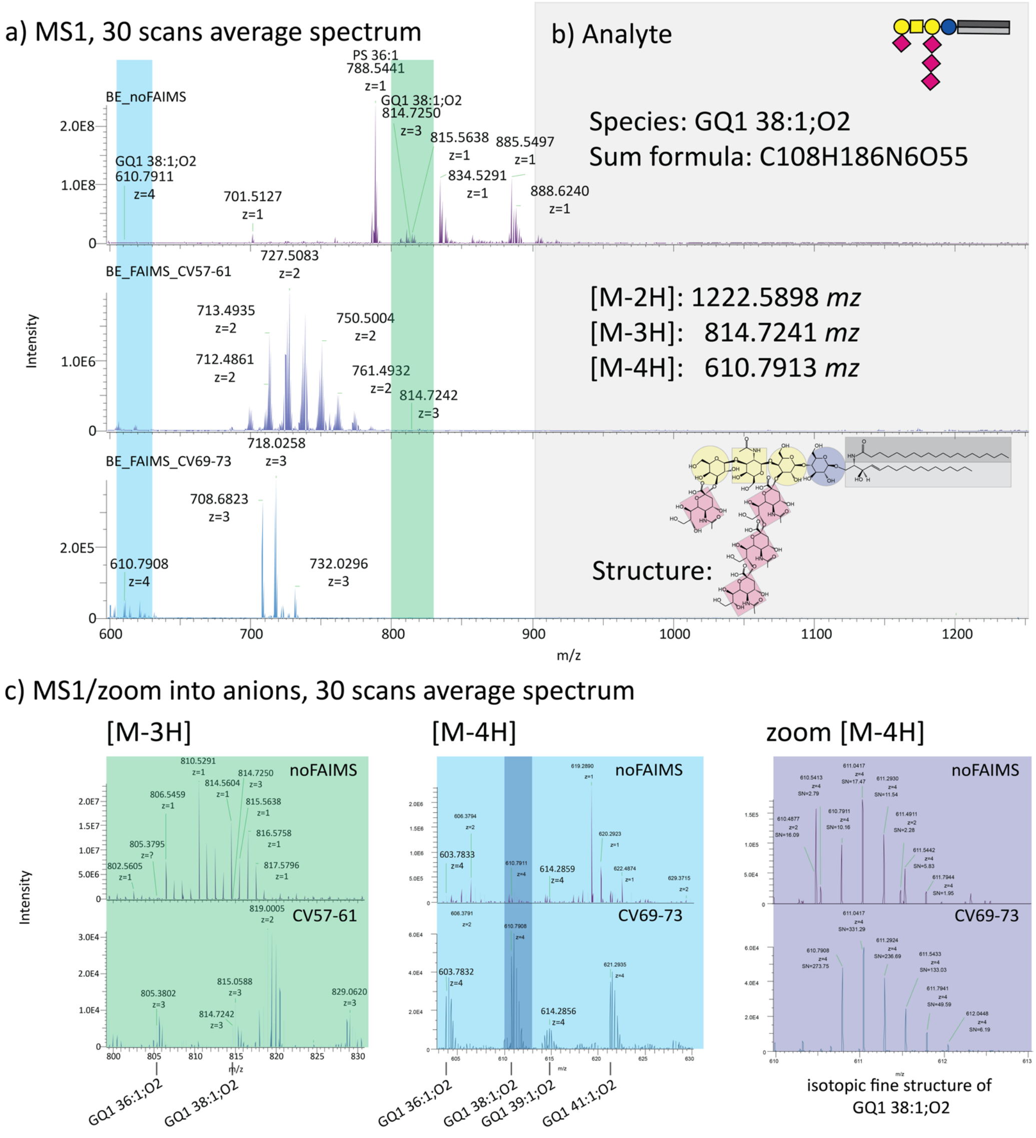


**Supplementary Figure 8.** Comparison of shotgun and FAIMS (both without AF) in the porcine brain extract (BE) on the example of GQ1 38:1;O2. (a) MS1 of shotgun (noFAIMS) and FAIMS at two different CV ranges for the detection of doubly (CV 57-61) or triply and quadruply (CV 69-73) charged gangliosides. A zoom (c) into *m/z* regions for triply charged GQ species (green), and quadruply charged GQ species (blue) demonstrates the improved ganglioside detection by FAIMS. An additional zoom in the isotopic fine structure of GQ1 38:1;O2 (violet) showcases the S/N improvements achieved by FAIMS, as overlapping signals of species at lower charge states are removed by the FAIMS source. The structure of GQ1 38:1;O2 is shown in (b).

Demonstrated at the example of GQ1 38:1;O2 (see also in Supplementary Figure 8 for MS1 info), shotgun FAIMS enabled the identification via MS/MS in the pooled ganglioside standard (PGS) (Supplementary Figure 11a and b). In the analysis of the complex porcine brain sample, the acquisition of MS/MS for GQ1 38:1;O2 was observed solely using the FAIMS setup (Supplementary Figure 11c). Despite the considerably low total signal intensity, resulting in a compromised spectral quality, the unambiguous identification was facilitated by the presence of diagnostic sialic acid fragments. Additionally, the assignment of the precursor to the MS/MS, leveraging DDA-based experiments, further contributes to the confidence in the identification process. This example highlights the beneficial characteristics of FAIMS for enhanced ganglioside detection and annotation.

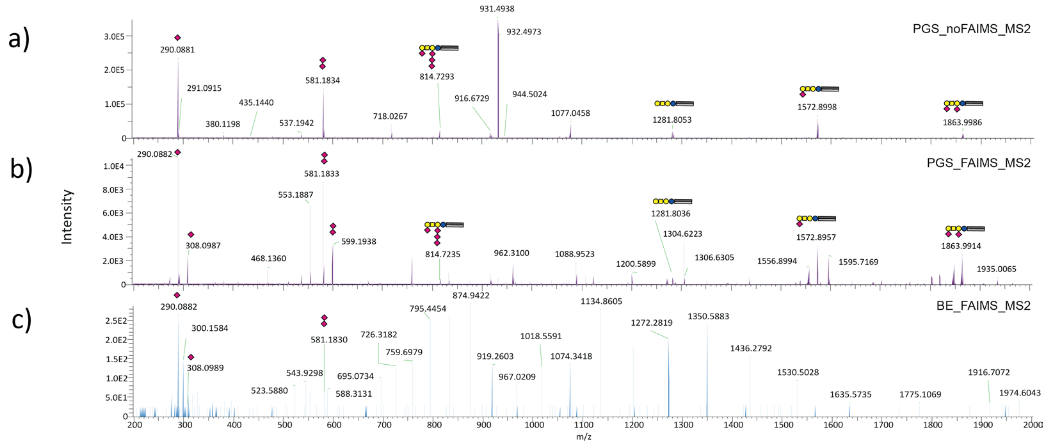


**Supplementary Figure 9.** Average MS/MS spectrum of GQ1 38:1;O2 combining CID, HCD and UVPD information in (a) the pooled ganglioside standard (PGS) with shotgun, (b) PGS using FAIMS, and (c) the porcine brain extract (BE) using FAIMS. All three MS/MS spectra were acquired in absence of AF.

Generally, FAIMS improved the initiation of MS/MS data acquisition, particularly for low-intensity ganglioside species and enhances the quality of fragment spectra by reducing overlaps on the MS/MS level (see Supplementary Figure 10). This enhancement significantly contributes to improved identification capabilities in complex sample analysis. Overall, FAIMS demonstrates its efficacy in improving both S/N and spectral quality, offering valuable applications in complex sample analyses, such as ganglioside annotation.

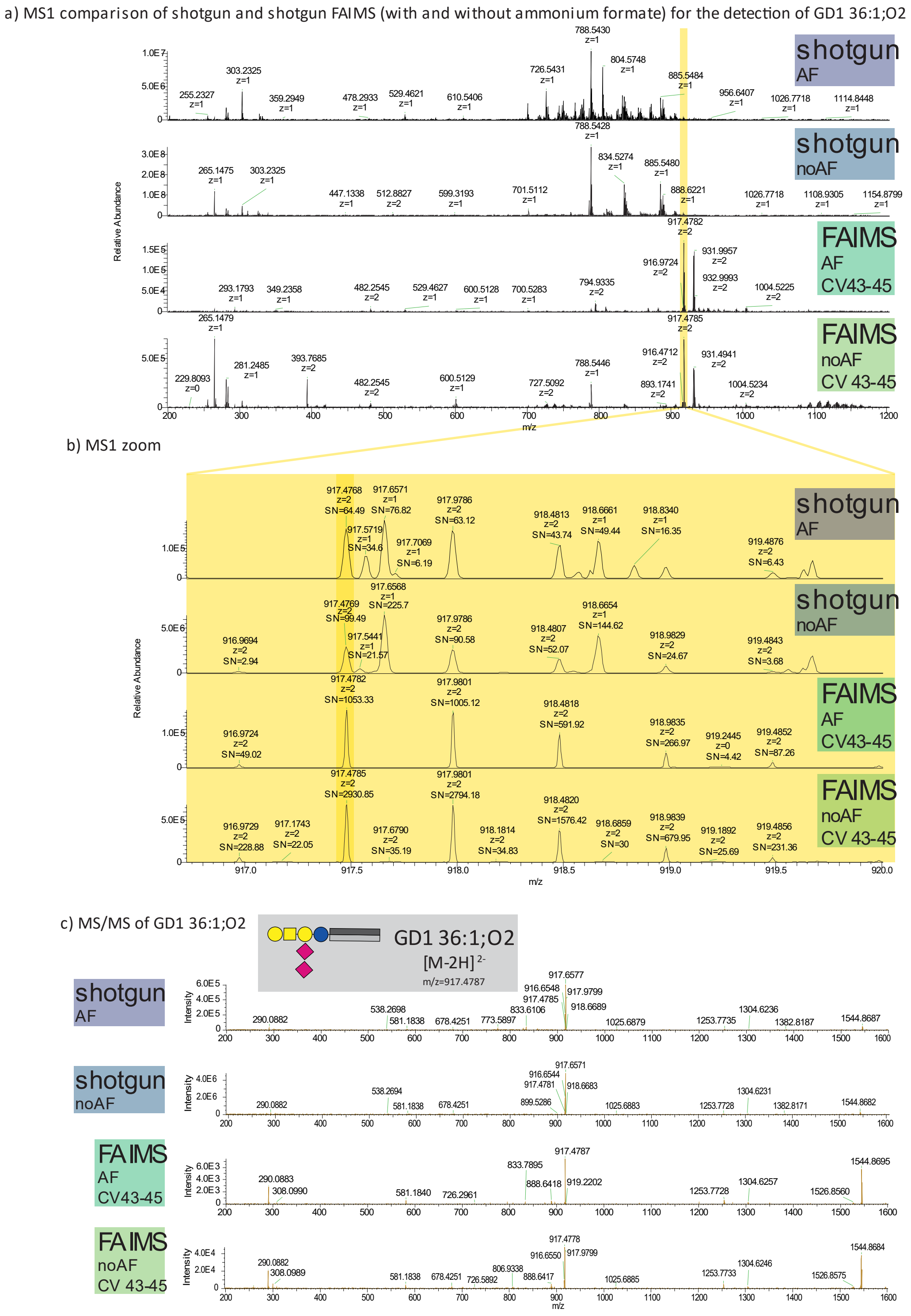


**Supplementary Figure 10.** (a) Comparison of shotgun and shotgun FAIMS (MS1) on the example of GD1 36:1;O2 in the presence or absence of AF. (b) FAIMS efficiently eliminates isobaric overlaps (singly negatively charged *m/z* 917.5 and 917.6) and thereby significantly improves the S/N ratio for the targeted doubly negatively charged *m/z* 917.4787 by a factor of approximately 30. (c) Comparison of MS/MS scans with and without FAIMS. In no FAIMS, several higher abundant species overlap with the signal of GD1 36: 1;O2 $[M-2H]^{2-}$ at *m/z* 917.4777. These undesired interfering species result in a hybrid MS/MS spectrum, where both species are co-i solated. By applying FAIMS, the signal of the singly charged interfering species can be demoted by using higher CVs, resulting in a clean MS/MS scan. Correspondingly, the spectral quality of low abundant gangliosides can be significantly improved by FAIMS. The presented data is acquired from the porcine brain extract analyzed on MS1, averaging 20 MS1 scans.

# 4 Extension: Enhancing automated ganglioside annotation with the LDA

Our method does not only provide a faster alternative to traditional HILIC methods but also eliminates the need for re-equilibration, making it more efficient and suitable for high-throughput analyses. Compared to traditional shotgun analyses, our FAIMS method offers additional separation dimensions, entailing cleaner MS/MS spectra and improving as such the identification rate, especially for multiply charged ganglioside species.

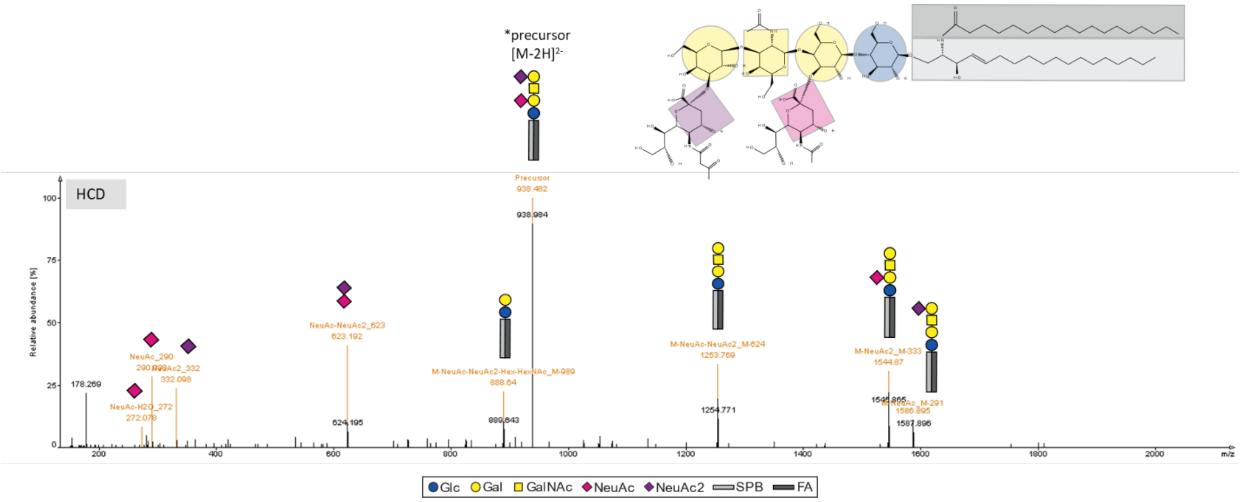


**Supplementary Figure 11.** Automated LDA annotation at the example of GD1-Ac 36:1;O2 in the porcine brain extract using FAIMS without the addition of ammonium formate (UVPD fragmentation). Diagnostic fragments NeuAc, NeuAc2, and NeuAc-NeuAc2 (NeuAc= sialic acid, NeuAc2 = acetylated sialic acid) have been detected, as well as their neutral losses (M-NeuAc, M-NeuAc2 and M-NeuAc-NeuAc2).

The LDA provides automated assignment of (molecular) ganglioside species in shotgun and shotgun FAIMS generated data files. An annotation example is shown in the Supplementary Figure 11, whereas Supplementary Figure 12 gives insights into the setup of a decision rule. The decision rules are used in the background along with a ganglioside specific mass list for the calculation of ganglioside species and their respective fragment for a defined ion species.

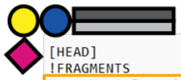

GM3 [M-H]$^-$ decision rules

```
[HEAD]
!FRAGMENTS
Name=NeuAcFrag_87              Formula=C3H3O3                                             Charge=1   MSLevel=2   mandatory=false
Name=NeuAc-H2O_272             Formula=C11H14NO7                                          Charge=1   MSLevel=2   mandatory=false
Name=Hex_161                   Formula=C6H9O5                                             Charge=1   MSLevel=2   mandatory=false
Name=Hex-O_179                 Formula=C6H11O6                                            Charge=1   MSLevel=2   mandatory=false
Name=NeuAc_290                 Formula=C11H16NO8                                          Charge=1   MSLevel=2   mandatory=true
Name=NeuAc-O_308               Formula=C11H18NO9                                          Charge=1   MSLevel=2   mandatory=false
Name=Hex-Hex_323               Formula=C12H19O10                                          Charge=1   MSLevel=2   mandatory=false
Name=Hex-Hex-O_341             Formula=C12H21O11                                          Charge=1   MSLevel=2   mandatory=false
Name=NeuAc-Hex_452             Formula=C17H26NO13                                         Charge=1   MSLevel=2   mandatory=false
Name=NeuAc-Hex-O_470           Formula=C17H28NO14                                         Charge=1   MSLevel=2   mandatory=false
Name=NeuAc-Hex-Hex_614         Formula=C23H36NO18                                         Charge=1   MSLevel=2   mandatory=false
Name=NeuAc-Hex-Hex-O_632       Formula=C23H38NO19                                         Charge=1   MSLevel=2   mandatory=false
Name=Precursor                 Formula=$PRECURSOR                                         Charge=2   MSLevel=2   mandatory=false
Name=PrecursorSingle           Formula=$PRECURSOR+H                                       Charge=1   MSLevel=2   mandatory=false
Name=M-Hex_M-162               Formula=PrecursorSingle-C6H10O5                            Charge=1   MSLevel=2   mandatory=false
Name=M-Hex-O_M-180             Formula=PrecursorSingle-C6H12O6                            Charge=1   MSLevel=2   mandatory=false
Name=M-NeuAc_M-291             Formula=PrecursorSingle-C11H17NO8                          Charge=1   MSLevel=2   mandatory=false
Name=M-NeuAc-O_M-309           Formula=PrecursorSingle-C11H19NO9                          Charge=1   MSLevel=2   mandatory=false
Name=M-Hex-Hex_M-324           Formula=PrecursorSingle-C12H20O10                          Charge=1   MSLevel=2   mandatory=false
Name=M-Hex-Hex-O_M-342         Formula=PrecursorSingle-C12H22O11                          Charge=1   MSLevel=2   mandatory=false
Name=M-NeuAc-Hex_M-453         Formula=PrecursorSingle-C17H27NO13                         Charge=1   MSLevel=2   mandatory=false
Name=M-NeuAc-Hex-O_M-471       Formula=PrecursorSingle-C17H29NO14                         Charge=1   MSLevel=2   mandatory=false
Name=M-NeuAc-Hex-Hex_M-615     Formula=PrecursorSingle-C23H37NO18                         Charge=1   MSLevel=2   mandatory=false
Name=M-NeuAc-Hex-Hex-O_M-633   Formula=PrecursorSingle-C23H39NO19                         Charge=1   MSLevel=2   mandatory=false

[CHAINS]
!FRAGMENTS
Name=FA_FA                     Formula=$CHAIN                                             Charge=1   MSLevel=2   mandatory=false
Name=FA-H_FA-1                 Formula=$CHAIN-H                                           Charge=1   MSLevel=2   mandatory=false
Name=FA-H2O_FA-18              Formula=$CHAIN-H2O                                         Charge=1   MSLevel=2   mandatory=false
Name=LCB-H2O_LCB-18            Formula=$LCB-H3O                                           Charge=1   MSLevel=2   mandatory=false
Name=LCB-2H2O_LCB-36           Formula=$LCB-H5O2                                          Charge=1   MSLevel=2   mandatory=false
Name=G                         Formula=PrecursorSingle-$CHAIN+CH2NO                       Charge=1   MSLevel=2   mandatory=false
Name=G-NeuAc_G-291             Formula=PrecursorSingle-$CHAIN+CH2NO-C11H17NO8             Charge=1   MSLevel=2   mandatory=false
Name=G-NeuAc-NeuAc_G-582       Formula=PrecursorSingle-$CHAIN+CH2NO-C11H17NO8-C22H34N2O16 Charge=1   MSLevel=2   mandatory=false

!INTENSITIES
Equation=G|G-NeuAc_G-291|G-NeuAc-NeuAc_G-582                                                                    mandatory=true
```

**Supplementary Figure 12.** LDA decision rule shown on the example of GM1 [M-H]$^-$. Fragments for the ganglioside (yellow box) as well as neutral losses (bright green box) are defined. Furthermore, fragments for the ceramide chain (dark green box) are defined. The highlighted yellow line demonstrates a mandatory=true fragment. This is a fragment, that must be present for a positive annotation. For most gangliosides classes, the diagnostic sialic acid fragment (NeuAc) is defined as mandatory=true fragment. Another mandatory=true fragment is the G-fragment (or G-NeuAc, or G-NeuAc-NeuAc), which is generated by UVPD fragmentation and must be present for the assignment of the molecular lipid species.

The LDA extension for the automated ganglioside (molecular) lipid species assignment in shotgun and shotgun FAIMS data includes a total of 29 ganglioside classes and modifications (fucosylated, acetylated): GM1, GM2, GM3, GM4, GM1-Ac, GM1-Fuc, GD1, GD2, GD3, GD1-Ac, GD1-Fuc, GD1-Fuc-Ac, GD3-Ac, GT1, GT2, GT3, GT1-Ac, GT1-Fuc, GT3-Ac, GT3-Fuc, GQ1, GQ1-Ac, GQ1-Fuc, GP1, GH1, GS1, GS1-Fuc, GO1. This covers ganglioside classes with 1 to 8 sialic acids attached to the glycan moiety. On the ceramide moiety a total of 30 to 50 carbon atoms, 0 to 6 double bonds as well as di- and tri-hydroxylated species are allowed. This leads to a total of around 8500 distinct ganglioside species that are available with the developed LDA extension.

Supplementary Table 6 (Benchmarking data set) and 7 (LDA data set) give an overview of the number of annotated ganglioside species for each ganglioside class in the pooled ganglioside standard (PGS) and the porcine total brain extract (BE). The presence or absence of ammonium formate (AF) as well as the method (shotgun=noFAIMS, shotgun FAIMS= FAIMS) are indicated. Additionally, the most abundant ion species is presented. A comprehensive listing of all 112 annotated ganglioside species in the pooled ganglioside standard and the porcine brain extract, detailing the main ion species detected and the method employed for species identification, is available in Supplementary Information ID Table.

**Supplementary Table 6.** Benchmarking data set. Total numbers of annotation for the pooled ganglioside standard (PGS) and the brain extract (BE)[a].

| Sample | GM1 | GM2 | GM3 | GD1 | GD2 | GD3 | GT1 | GT2 | GT3 | GQ1 | GP1 | IDs | Total |
|---|---|---|---|---|---|---|---|---|---|---|---|---|---|
| PGS/AF/noFAIMS | 4 | 2 | 19 | 7 | 11 | 20 | 5 | 2 | 0 | 0 | 0 | 70 | 112 |
| PGS/noAF/noFAIMS | 2 | 1 | 16 | 7 | 11 | 26 | 5 | 2 | 0 | 3 | 2 | 75 | |
| PGS/AF/FAIMS | 4 | 2 | 12 | 13 | 15 | 30 | 12 | 4 | 0 | 5 | 2 | 99 | |
| PGS/noAF/FAIMS | 4 | 2 | 13 | 13 | 15 | 30 | 12 | 4 | 5 | 6 | 2 | 106 | |
| BE/AF/noFAIMS | 2 | 0 | 0 | 7 | 2 | 9 | 5 | 0 | 0 | 0 | 0 | 25 | 44 |
| BE/noAF/noFAIMS | 2 | 0 | 0 | 7 | 2 | 10 | 5 | 0 | 0 | 3 | 0 | 29 | |
| BE/AF/FAIMS | 2 | 0 | 0 | 11 | 2 | 10 | 11 | 0 | 0 | 3 | 0 | 39 | |
| BE/noAF/FAIMS | 2 | 0 | 0 | 11 | 2 | 10 | 11 | 0 | 4 | 4 | 0 | 44 | |
| Ion Species | [M-H]$^-$ | [M-H]$^-$ | [M-H]$^-$ | [M-2H]$^{2-}$ | [M-2H]$^{2-}$ | [M-2H]$^{2-}$ | [M-3H]$^{3-}$ | [M-2H]$^{2-}$ [M-3H]$^{3-}$ | [M-3H]$^{3-}$ | [M-3H]$^{3-}$ [M-4H]$^{4-}$ | [M-4H]$^{4-}$ [M-5H]$^{5-}$ | | |

[a]The numbers are demonstrated for different ganglioside classes along with the main ion species detected.

**Supplementary Table 7.** LDA data set. Total numbers of automatically generated annotation (no further data processing) for the pooled ganglioside standard (PGS) and the brain extract (BE)[a].

| Sample | GM1 | GM2 | GM3 | GM4 | GD1 | GD1-Ac | GD2 | GD3 | GT1 | GT2 | GT3 | GQ1 | GS1-Fuc | GO1 | IDs | Total |
|---|---|---|---|---|---|---|---|---|---|---|---|---|---|---|---|---|
| PGS/AF/noFAIMS | 5 | 4 | 14 | 1 | 18 | 1 | 10 | 23 | 14 | 5 | 0 | 4 | 2 | 4 | 105 | 109 |
| PGS/noAF/noFAIMS | 5 | 4 | 14 | 1 | 18 | 1 | 10 | 23 | 14 | 5 | 1 | 4 | 3 | 5 | 108 | |
| PGS/AF/FAIMS | 5 | 4 | 14 | 1 | 18 | 1 | 10 | 23 | 14 | 5 | 0 | 4 | 2 | 4 | 105 | |
| PGS/noAF/FAIMS | 5 | 4 | 14 | 1 | 18 | 1 | 10 | 23 | 14 | 5 | 1 | 4 | 4 | 5 | 109 | |
| BE/AF/noFAIMS | 5 | 3 | 5 | 0 | 13 | 1 | 0 | 7 | 7 | 4 | 0 | 0 | 0 | 1 | 46 | 107 |
| BE/noAF/noFAIMS | 1 | 2 | 0 | 0 | 14 | 1 | 1 | 15 | 14 | 1 | 1 | 4 | 0 | 1 | 55 | |
| BE/AF/FAIMS | 5 | 4 | 3 | 1 | 18 | 1 | 5 | 18 | 14 | 5 | 1 | 3 | 2 | 3 | 83 | |
| BE/noAF/FAIMS | 4 | 2 | 13 | 1 | 18 | 1 | 10 | 22 | 14 | 5 | 1 | 4 | 4 | 5 | 104 | |
| Ion Species | [M-H]$^-$ | [M-H]$^-$ | [M-H]$^-$ | [M-H]$^-$ | [M-2H]$^{2-}$ | [M-2H]$^{2-}$ | [M-2H]$^{2-}$ | [M-2H]$^{2-}$ | [M-3H]$^{3-}$ | [M-2H]$^{2-}$ [M-3H]$^{3-}$ | [M-3H]$^{3-}$ | [M-3H]$^{3-}$ [M-4H]$^{4-}$ | [M-4H]$^{4-}$ [M-5H]$^{5-}$ | [M-4H]$^{4-}$ [M-5H]$^{5-}$ | | |

[a]The numbers are demonstrated for different ganglioside classes along with the main ion species detected.
