# Supplementary material for: FAIMS Shotgun Lipidomics for Enhanced Class- and Charge-State Separation Complemented by Automated Ganglioside Annotation
Source: Anal Chem. 2024 Jul 19;96(30):12296–307. doi: 10.1021/acs.analchem.4c01313 (PMC11295132; doi:10.1021/acs.analchem.4c01313)
Supplement: Supplementary file 3 — ac4c01313_si_003.pdf [file ac4c01313_si_003.pdf]

Data Availability

All study data, including

(1) shotgun and FAIMS raw data
(2) raw data as .zip folder
(3) LDA results as .zip folder

are available from GNPS using following link and password:

- https://massive.ucsd.edu/ProteoSAFe/dataset.jsp?accession=MSV000095088

Sample labeling description:
Sample_Concentration_Solvent_Polarity_Setup_MSmethod_SamplePosition_Replicate_Injection.raw
BE_250ugml_421AF_neg_FAIMS_auto5MS2_E5_1_a.raw
