# Supplementary material for: FAIMS Shotgun Lipidomics for Enhanced Class- and Charge-State Separation Complemented by Automated Ganglioside Annotation
Source: Anal Chem. 2024 Jul 19;96(30):12296–307. doi: 10.1021/acs.analchem.4c01313 (PMC11295132; doi:10.1021/acs.analchem.4c01313)
Supplement: Supplementary file 4 — ac4c01313_si_004.pdf [file ac4c01313_si_004.pdf]

Installation and Download Instructions for Lipid Data Analyzer 2.10.0

Table of Contents

1 Download Instructions 1
2 Installation Instructions..2
2.1 LDA download and installation using Microsoft Edge 2
3 Data review and visualization 5
4 Starting LDA 7

1 Download Instructions

Installers for LDA 2.10.0 can be downloaded using the following link:
http://genome.tugraz.at/lda2/

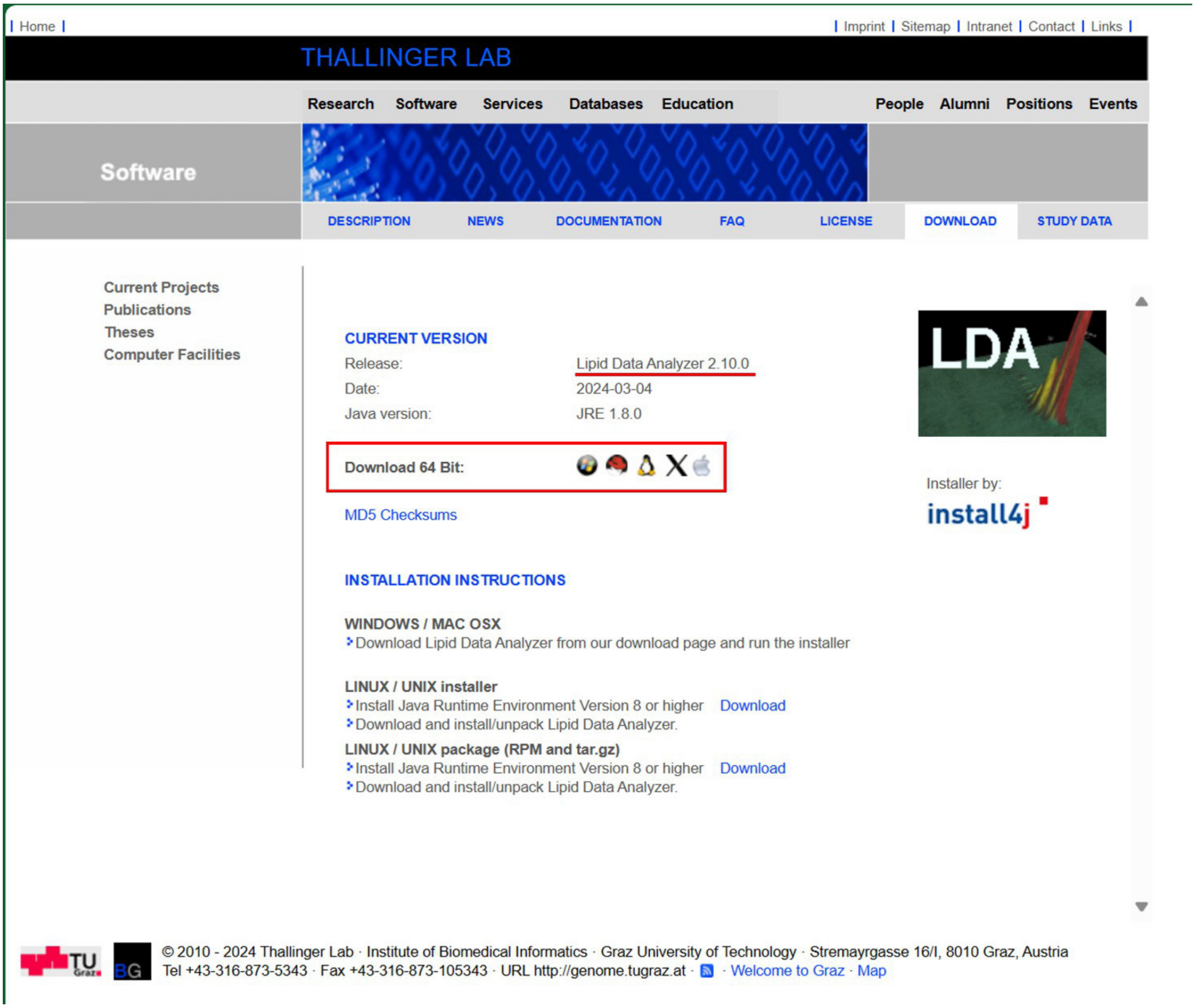

2 Installation Instructions

Installers for LDA are available for Windows, Linux/Unix and Mac OSX. For Windows, LDA is bundled with a tested Java JRE, and includes msconvert to directly use raw data from AB Sciex, Agilent Technologies, Bruker Daltonics and Thermo Fisher Scientific. For Windows, there is no need for installing additional software packages. It is sufficient to simply run the installer. Windows is the recommended operating system for using LDA, and the detailed installation instructions provided in this manual are taken from the Windows installation procedure.
As for Windows, LDA installation for Mac OSX does not require any additional software packages. However, this installer does not provide the vendor libraries, and the latest JRE provided by Mac OSX has not been tested yet. Thus, it is advisable to install a JRE 1.8 (see below).
For LINUX/UNIX, JRE 1.8 has to be installed (if not already done), which can be downloaded from here:
https://www.oracle.com/java/technologies/javase-downloads.html
For the LDA installation at LINUX/UNIX platforms, the following options are available:

- Installer using a shell script (the bash script must be given executable rights)
- An RPM package
- A tar.gz archive

2.1 LDA download and installation using Microsoft Edge

To start the download, click on the Windows icon at the LDA download page (1). Upon completion of the download, a warning that the software might be potentially harmful will be shown by Microsoft Edge (2). Click on the three dots next to the warning (3) and select ‘Keep’ (4).

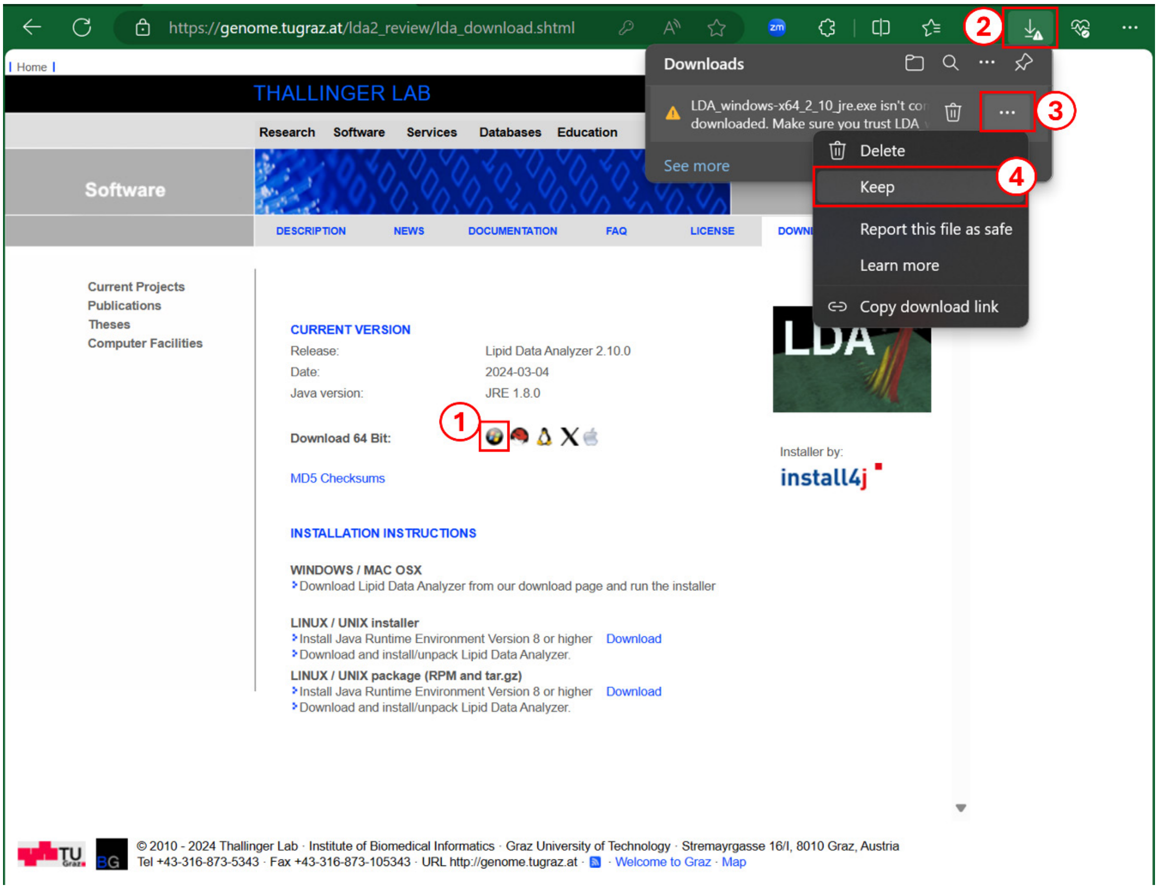

Again, Microsoft Edge will ask whether you are sure to keep this file. To do so, click on 'Show more' (5), and select 'Keep anyway' (6):

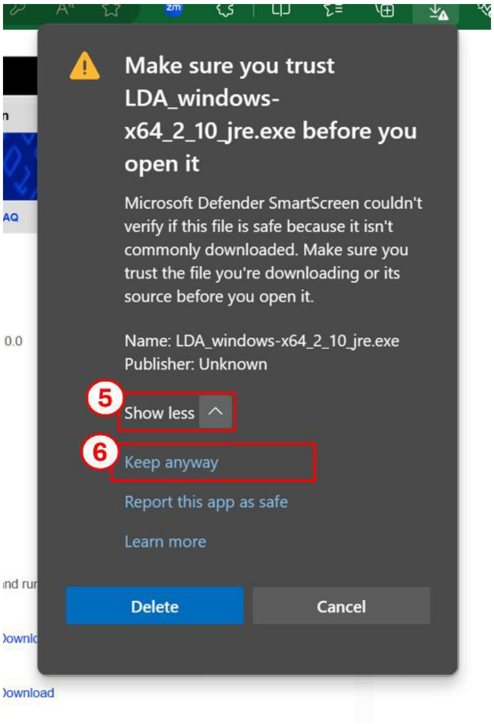

The installer called 'LDA_windows-x64_2_10_jre.exe' will then be located in the 'Downloads' directory. To start the installation, execute LDA_windows-x64_2_10_jre.exe with administrative privileges.
The installer will ask for some information such as the installation directory. Despite the straightforward nature of most responses in the installation process, three dialogues require further attention:

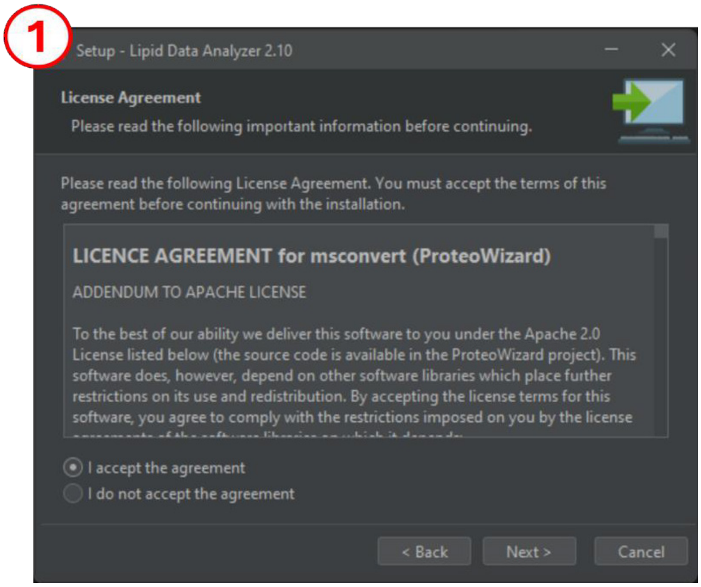

(1) During the Windows installation process, the installer will prompt users to consent to several licenses, as LDA includes the independent software package msconvert as well as vendor libraries essential for raw data conversion. It is necessary to provide consent for all agreements presented during the installation process.
(2) After selecting the installation directory, LDA will provide you with a selection of MS instruments. It does not matter which instrument you select during the installation process. LDA provides many more settings for instruments than shown in this selection. Simply click on ‘Next >’ at this step.

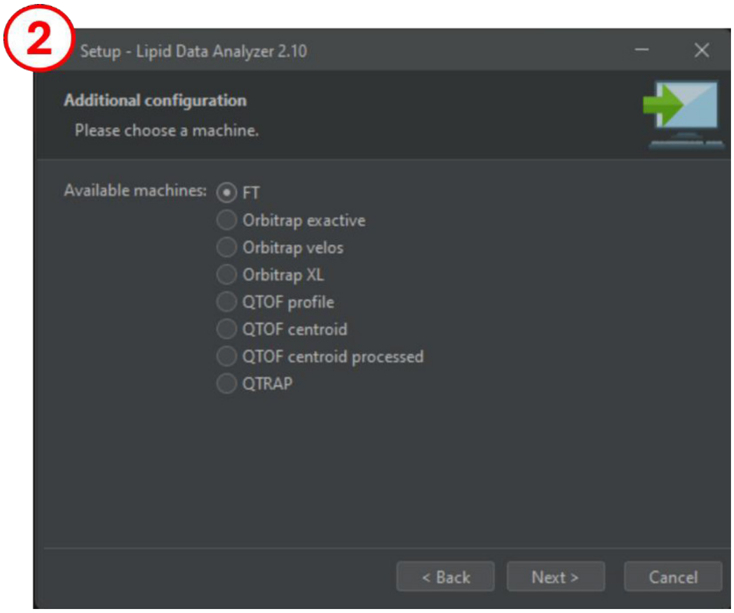

(3) The final question of the installer will be the memory settings:

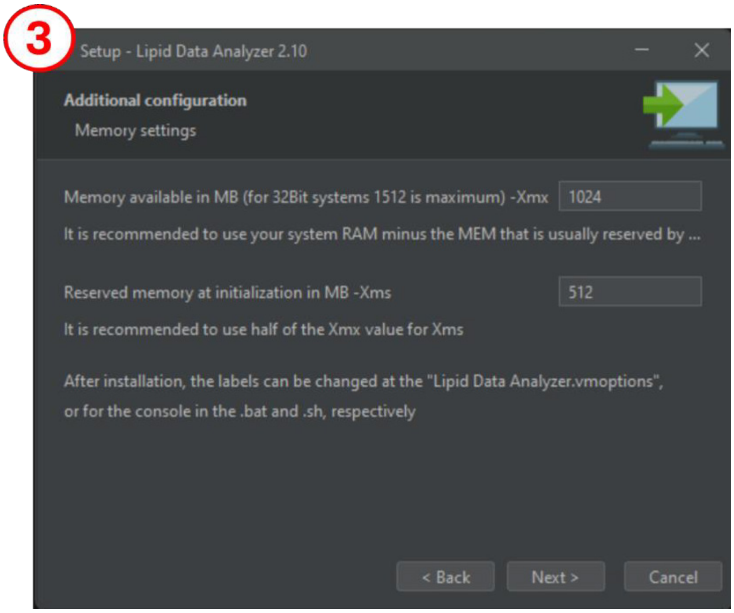

Java does not reserve more memory on your PC/laptop than you specify by those settings to avoid crashes of your system. The values provided are in MB (1 GB corresponds to 1024 MB). The -Xmx value specifies the

maximum amount of memory that can be reserved by Java. When LDA would require more memory than this threshold value, it will crash, and you will see a java.lang.OutOfMemoryError. The -Xms value specifies the memory of LDA when it starts. If you have a PC with 2 GB or less memory, keep the settings as they are. Otherwise, it is recommendable to increase those memory settings.
For -Xms, typically 1024 is sufficient.
For -Xmx it is recommended to use 2 GB less than the system memory. For instance, if the system has 16 GB of RAM, it is advisable to set -Xmx to 14 GB (in this case the entered value should be 14 336, which is 14 times 1024).
Additional information regarding these settings and how to change them after the installation can be found at question 4 of the FAQ and at the following PDF:
http://genome.tugraz.at/lda2/lda_faq.shtml
Furthermore, an informative resource for expediting the conversion of raw files to the LDA-specific chrom file format can be accessed via the following link:
http://genome.tugraz.at/lda2/ChromTranslationAcceleration.pdf

3 Data review and visualization

In order to visualize data of a single MS run, start the LDA, click on "Settings" tab, select OrbiTrap_Lumos_shotgun, select Ganglioside_neg_shotgunFAIMS, and click on "Save as default":

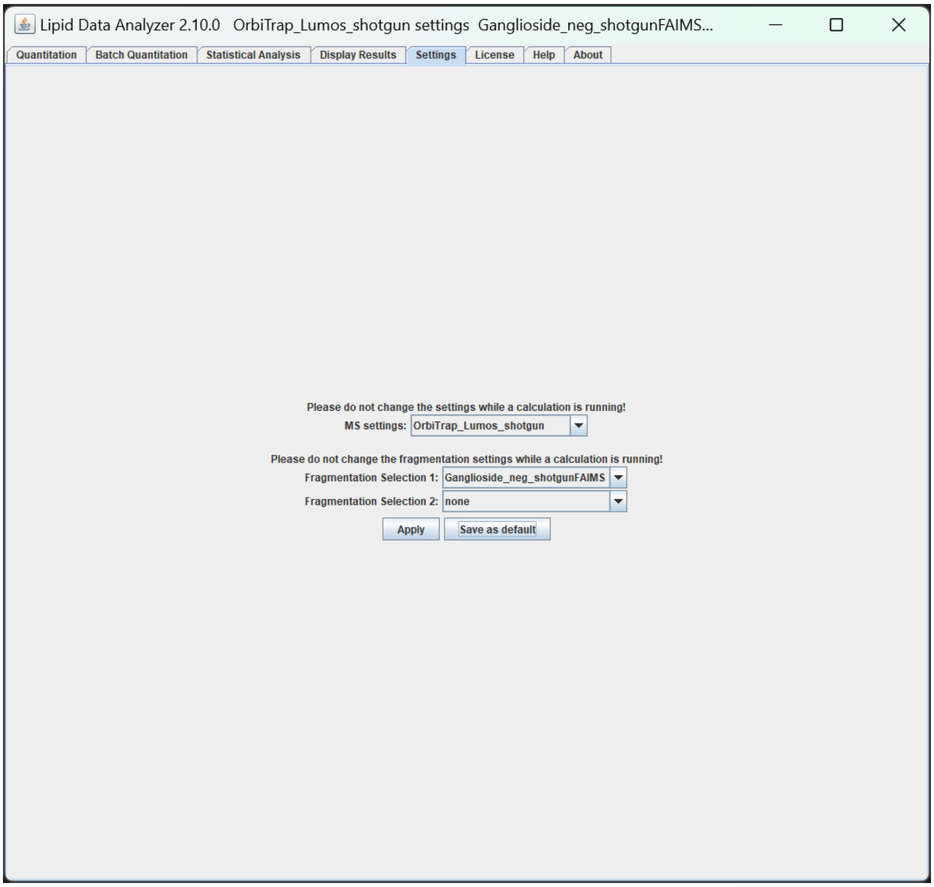

To visualize the project data, navigate to the "Display Results" tab and choose the relevant LDA results, specifically the chrom file and its corresponding LDA Excel result file. Then click on “Start Display”.
A menu on the left side will appear, now containing a selection box at the top, which can be used to switch between the different lipid subclasses. Then a table follows, whereupon the name of the analyte is in the first column, and the area in the second.
For shotgun data, the display name consists of “$name$:$double_bonds$_$ion_species$” (e.g. d38:1_-H2).

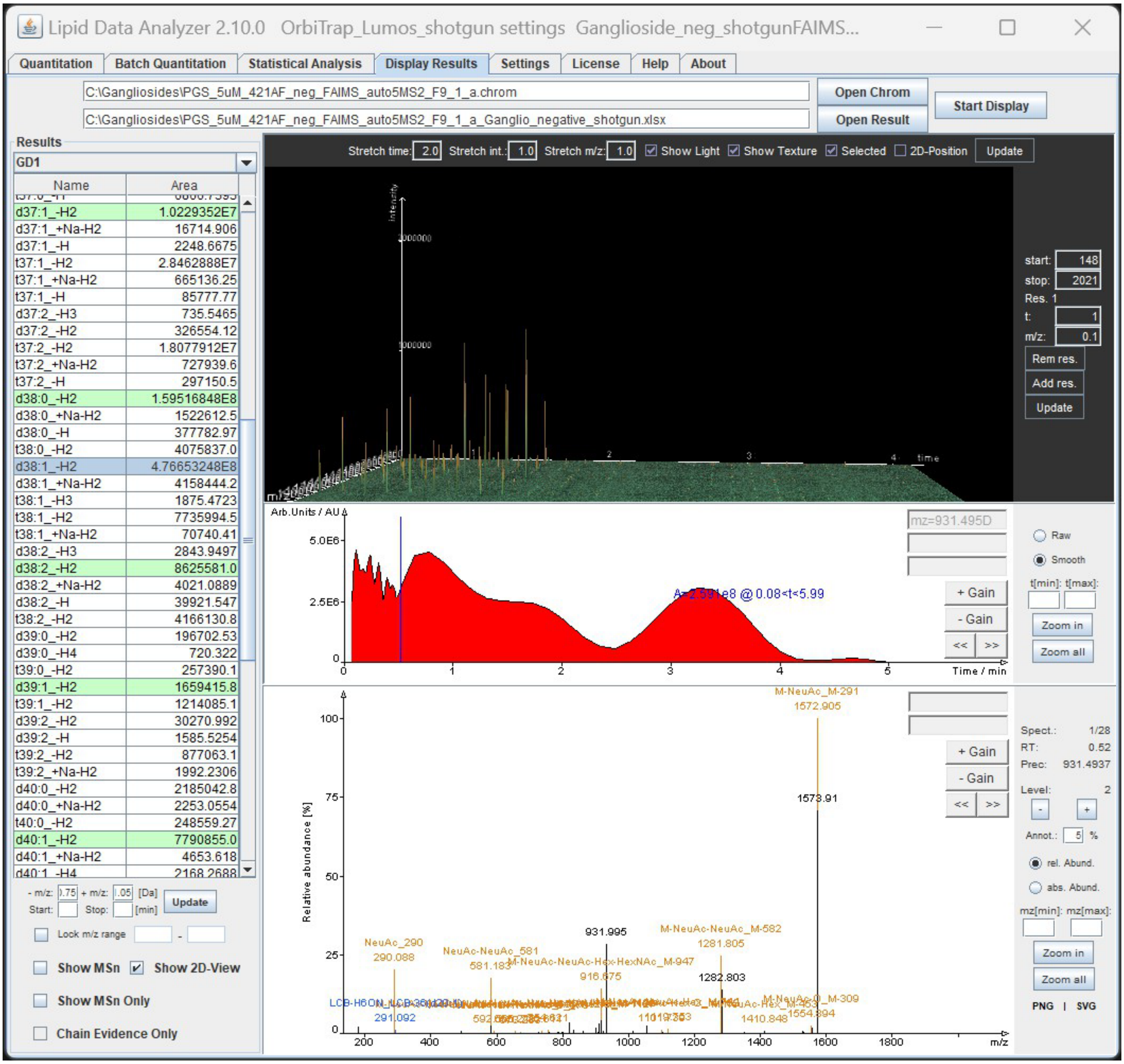

The color encoding of the table entries has the following meaning:

- White: MS^1 identification without any MS^n evidence.
- Green: MS^1 identification verified by MS^n spectra.
- Blue: Current selection for display.

The “Show MSn” checkbox switches from the lipid species to the lipid molecular species view of the table where possible.

Now, the table displays information about the obtained structure according to Liebisch, G. et al. Shorthand notation for lipid structures derived from mass spectrometry. J Lipid Res 54, 1523-1530 (2013). I.e., the sum of carbon atoms and double bonds in the chains if no structural information can be obtained (e.g. 38:4), the fatty acid chains separated by an underscore if fatty acids are detectable (e.g. 20:4_18:0), or the fatty acid chains separated by a slash if the position of the fatty acid chains is assignable (e.g. 18:0/20:4).
With "Show MSn Only" selected, only MS^n identifications are displayed and with "Chain Evidence Only", only those MS^n identifications with evidence for the identity of the fatty acid chains are displayed.

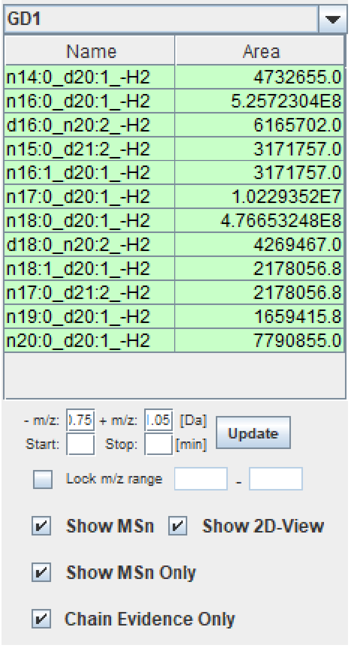

Attention: For the manual inspection of MS^n, there is a difference in the displayed annotation of the spectra, if the lipid species consists of more than one lipid molecular species:

- The lipid species view (available when "show MSn" is NOT checked) is selected: The fragments of all detected lipid molecular species are annotated.
- The lipid molecular species view (available when "show MSn" is checked) is selected: Only the fragments of the selected lipid molecular species are annotated.

When a row of the table is activated by the left mouse button, the peaks are displayed in the 3D and the chromatogram viewer. When a row is activated by the right mouse button, a popup appears.
By clicking "Show MS/MS", MS/MS spectra are shown as annotated by the used MS^n decision rules at the time of quantitation.

If the reviewer wants to reproduce the obtained results, the required mass list can be found within the LDA installation directory (exampleMassList/gangliosides_FAIMS_shotgun). A detailed description of how to start a Batch Quantitation can be found in the accompanying LDA user manual (see help tab of LDA software).

4 Starting LDA

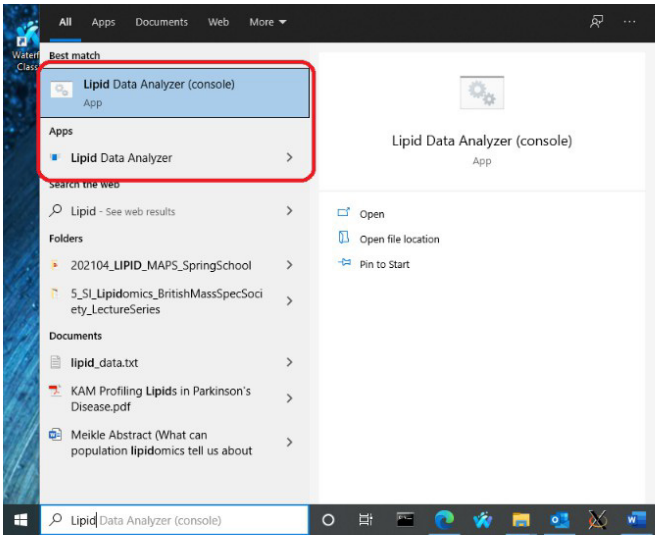

There are two ways to start LDA (the required files for both options are located in the root of the installation folder of LDA). The first one is a conventional application ('Lipid Data Analyzer.exe' in Windows), while the second method launches a console window ('Lipid Data Analyzer.bat' in Windows – see Appendix D of the LDA user manual http://genome.tugraz.at/lda2/2.10/LDA_2.10.pdf).
Both versions can be located using the Windows application search function.
The recommendation is to opt for the latter choice, as it provides additional progress and troubleshooting information. Additionally, the absence of errors in the console output suggests that LDA requires more time to complete the ongoing task.
